# Immunohistochemical, Immunofluorescent, and Molecular Investigation of Canine Distemper Virus, Nectin 4, and CD150 Receptors in Naturally Infected Dogs with Canine Distemper Virus

**DOI:** 10.3390/ani16142233

**Published:** 2026-07-18

**Authors:** Hilmi Nuhoğlu, Enver Beytut

**Affiliations:** Department of Pathology, Faculty of Veterinary Medicine, Kafkas University, Kars 36100, Türkiye; enverbeytut@hotmail.com

**Keywords:** canine distemper virus, CD150, immunofluorescence, immunohistochemistry, nectin-4

## Abstract

Canine distemper virus uses CD150 and Nectin-4 receptors to infect cells. In the central nervous system, astrocytes, the main canine distemper virus target, reportedly lack these receptors, suggesting alternative entry pathways. This study investigated canine distemper virus distribution and receptor usage in naturally infected dogs’ organs and tissues using immunohistochemistry and immunofluorescence. Tissue samples from 34 dogs (including the central nervous system, internal organs, footpads, and planum nasale) were examined for CDV, CD150, Nectin-4, and GFAP; diagnosis was confirmed by immunochromatographic assay and RT-PCR. Thirty-two dogs were canine distemper virus positive. Triple immunofluorescence staining showed epithelial cells were CDV/Nectin-4 positive, lymphocytes and macrophages were CDV/CD150 positive, while astrocytes were CDV-positive but negative for both receptors. Double immunofluorescence for CDV and GFAP in the CNS confirmed astrocyte infection. These findings indicate that CDV enters lymphocytes/macrophages via CD150 and epithelial cells via Nectin-4, whereas astrocytes likely use alternative entry pathways outside the classical receptor framework.

## Simple Summary

Canine distemper virus (CDV) causes a severe and often fatal disease in dogs. To infect a dog’s body, the virus uses specialized structures known as CD150 and Nectin-4 receptors to enter cells. Scientists already know that the virus uses these receptors to enter immune cells and cells lining various organs. However, how the virus enters some brain cells that appear to lack these specific receptors is still unclear. In this study, we examined tissue samples from naturally infected dogs to observe how the virus spreads. We found that CDV target cells are associated with CD150 expression in lymphoid cells and Nectin-4 expression in epithelial tissues. Importantly, we found that some supporting brain cells (astrocytes), despite being heavily infected by the virus, lacked both of these known receptors. This finding suggests that the virus may utilize an alternative pathway distinct from the classical CD150 and Nectin-4 receptors during central nervous system invasion.

## 1. Introduction

Canine distemper (CD) is a viral disease commonly found in carnivores worldwide. The causative agent is a *Morbillivirus* belonging to the *Paramyxoviridae* family. CDV is found in all carnivores, primarily dogs, as well as in wild cats. The canine distemper virus causes a multisystemic infection affecting the respiratory, digestive, and urinary systems, as well as the skin and, most notably, the central nervous system (CNS). The disease is associated with very high morbidity and mortality rates [1,2,3,4,5,6,7]. Like other morbilliviruses, CDV uses the Signaling Lymphocyte Activation Molecule (SLAM, SLAMF1, or CD150) and Nectin-4 receptors to enter cells. The virus infects lymphocytes and macrophages via the CD150 receptor and epithelial cells via the Nectin-4 receptor [8,9]. Although astrocytes are the most commonly infected cells in the central nervous system, neither of these two receptors is present in astrocytes [10]. Therefore, while it is hypothesized that there may be different pathways that the virus uses to enter astrocytes, these pathways have not yet been identified [11,12,13].

In this study, systemic necropsies were performed on 34 dogs of various ages and breeds diagnosed with distemper, and samples were collected from the lungs, heart, thymus, stomach, segments of the small intestine, mesenteric, mediastinal, and tracheobronchial lymph nodes, pancreas, liver, spleen, kidneys, bladder, brain, cerebellum, brainstem, spinal cord, footpads, and planum nasale. Tissue samples were examined for CDV, CD150, Nectin-4, and Glial Fibrillary Acidic Protein (GFAP) in the CNS using IHC and IF. Furthermore, CDV infection was evaluated using immunochromatography (IC) and Reverse-Transcriptase Polymerase Chain Reaction (RT-PCR), and these results were compared with IHC findings.

## 2. Materials and Methods

### 2.1. Animal Material

Permission for this study was obtained from the Local Ethics Committee for Animal Experiments at Kafkas University via Decision No. KAÜ-HADYEK 2021-045 dated 21 March 2021, confirming compliance with ethical guidelines. A total of 34 dogs of different ages, breeds, and sexes, which had died from canine distemper and were submitted to the Department of Pathology at the Faculty of Veterinary Medicine for necropsy, were used as study material. These dogs were obtained from the Kars Municipality Animal Shelter. Of these dogs, 18 were female, 11 were male, and the sex of five was not recorded. Of the 34 cases, 14 consisted of puppies with a history of early maternal separation and surrogate rearing, leading to suspected inadequate passive antibody transfer. Of the 34 dogs used as study material, 23 were identified as positive based on immunochromatographic (IC) test results and included in the study, while tissue samples for the remaining 11 cases were obtained from archived material. Additionally, five dogs of various breeds and ages that died from different causes, had no history of CDV in their medical records, and showed no symptoms were used as controls in the study.

### 2.2. Methods

This study involved recording the dogs’ medical histories, followed by testing nasal and ocular swab samples for CDV antigen using the IC method with the Asan Canine Distemper Rapid Diagnostic Kit (Code No: 022321; Asan Pharm. Co., Ltd., Anseong-si, Gyeonggi-do, Republic of Korea), in accordance with the kit’s user manual. Cases that tested positive by the IC method, as well as dogs exhibiting clinical respiratory problems (coughing, difficulty breathing, etc.), nasal and ocular discharge, and neurological symptoms (convulsions, tics, etc.), were accepted as material even if their IC tests were negative. From dogs that underwent systemic necropsies, samples were collected from the lungs, mediastinal and tracheobronchial lymph nodes, heart, thymus, stomach, small intestines, mesenteric lymph nodes, pancreas, liver, spleen, kidneys, bladder, brain, cerebellum, brainstem, spinal cord, footpads, and planum nasale.

#### 2.2.1. RT-PCR

Tissue samples were collected from the lungs, thymus, liver, stomach, intestine, mesenteric lymph node, spleen, cerebellum, kidney, and footpad to detect viral nucleic acid. The samples were processed, pooled, and nucleic acid extraction was performed using phenol-chloroform [14]. Since the CDV genome consists of RNA, a reverse transcription step was performed to synthesize complementary DNA (cDNA) prior to PCR amplification. For this purpose, the primer selected for the nucleoprotein region [15] was used in accordance with the manufacturer’s instructions for the 2× One-step RT-PCR master mix (HibriGen Biotechnology, Istanbul, Türkiye) kit. An extract that was previously sequenced from previous studies was used as positive control and molecular grade water was used as negative control. The resulting products were subjected to electrophoresis at 100 V on a gel prepared with ethidium bromide and visualized under UV light.

#### 2.2.2. Hematoxylin-Eosin (H&E)

Tissue samples obtained from the organs and tissues of dogs with distemper and control dogs were fixed in a 10% neutral buffered formalin solution for 24–48 h, then processed through routine histological procedures to create paraffin blocks. Sections 4–5 µm thick were cut from the paraffin blocks, stained with routine H&E, mounted with a mounting medium, coverslipped, and examined under a Olympus BX53 light microscope (Olympus Corporation, Tokyo, Japan).

#### 2.2.3. Immunohistochemistry (IHC)

Immunohistochemical staining was performed on serial sections taken from paraffin blocks and mounted on poly-L-lysine-coated slides using commercial antibodies against CDV, Nectin-4, CD150, and GFAP. After deparaffinization and hydration of the sections, the sections were incubated with 3% H_2_O_2_ for 20 min, then transferred to Citrate Buffer (pH 6.0) and boiled in a microwave oven (800 W) for 10 min. For IHC staining, the sections were incubated with 1:100 normal goat serum (30 min) as a blocking solution for anti-rabbit and anti-mouse primary antibodies, followed by the UltraVision Detection System Large Volume Anti-Polyvalent, HRP (TP-125-HL) staining kit (Thermo Fisher Scientific, Runcorn, UK) (30 min). Additionally, for Nectin-4 staining, the sample was incubated in a 1:100 normal rabbit serum blocking solution (30 min). In the performed staining experiments, Monoclonal Mouse anti-CDV antibody (Cat. No. MA1-82327; Thermo Fisher Scientific, Waltham, MA, USA), Human Nectin 4 Polyclonal Antibody (Cat. No. AF2659; R&D Systems, Minneapolis, MN, USA), Recombinant Anti-SLAM/CD150 Antibody (Cat. No. 10837-R008; Sino Biological Inc., Beijing, China), and Rabbit Anti-Glial Fibrillary Acidic Protein (GFAP) Polyclonal Antibody (Cat. No. AB5804; EMD Millipore, Temecula, CA, USA) were used as unlabeled primary antibodies. For the CDV, CD150, and GFAP primary antibodies, the Thermo UltraVision Detection System Large Volume Anti-Polyvalent, HRP (TP-125-HL) was used; for the Nectin-4 primary antibody, a biotinylated Rabbit anti-Goat IgG (H+L) secondary antibody (Cat. No. 31732; Invitrogen, Thermo Fisher Scientific, Waltham, MA, USA) was used. Subsequently, streptavidin from the Thermo HRP kit was used [16], (Table 1).

#### 2.2.4. Immunofluorescence

Both double and triple immunofluorescence (IF) staining techniques were applied in the immunofluorescence experiments. In triple staining, commercial primary antibodies against CDV, CD150, and Nectin-4 were used, along with corresponding fluorescently labeled secondary antibodies. In double IF staining, staining was performed using commercial primary antibodies against CDV and GFAP, along with appropriately labeled fluorescent secondary antibodies. In IF staining, the following were used: (Monoclonal Mouse anti-CDV (Cat. No. MA1-82327; Thermo Fisher Scientific, Waltham, MA, USA); Human Nectin-4 Polyclonal Antibody (Cat. No. AF2659; R&D Systems, Minneapolis, MN, USA); Recombinant Anti-SLAM/CD150 Antibody (Cat. No. 10837-R008; Sino Biological Inc., Beijing, China); and Rabbit Anti-Glial Fibrillary Acidic Protein (GFAP) Polyclonal Antibody (Cat. No. AB5804; EMD Millipore, Temecula, CA, USA) were used as unlabeled primary antibodies. Normal goat and donkey serum diluted 1:100 was used as the blocking solution. Fluorescently labeled secondary antibodies were used: Donkey anti-Goat IgG (H+L) Secondary Antibody, Alexa Fluor 350 (Cat. No. A21081; Invitrogen, Thermo Fisher Scientific, Waltham, MA, USA); Goat anti-Rabbit IgG (H+L) Secondary Antibody, Alexa Fluor 488 (Cat. No. A-11008; Invitrogen, Thermo Fisher Scientific, Waltham, MA, USA), and Goat anti-Mouse IgG (H+L) Secondary Antibody, Alexa Fluor 555 (Cat. No. A-21422; Invitrogen, Thermo Fisher Scientific, Waltham, MA, USA) were used. Under a fluorescence microscope, the Nectin-4 receptor appeared blue, the CD150 receptor and GFAP appeared green, and CDV appeared red. The identified microscopic fields were photographed using different filters without changing the fields of view. The images taken for each marker were overlaid using Adobe Photoshop CC 2019 (Adobe Systems Inc., San Jose, CA, USA), revealing the reactions produced by the antibodies in the same field and on the same cells (Table 1).

#### 2.2.5. Overlaying Immunofluorescence and Hematoxylin-Eosin Images

The sections that had been immunofluorescently stained and photographed were placed in distilled water again to remove the coverslips. After coverslip removal, the sections underwent routine H&E staining, starting with the hematoxylin step. For the same H&E-stained sections, the previously recorded coordinates of the areas photographed during IF staining were re-adjusted under the light microscope, and H&E photographs of the same areas were taken. The photographs obtained from the H&E staining were overlaid with the photographs taken at the same coordinates from the IF staining of these sections using Adobe Photoshop CC 2019 (Adobe Systems Inc., San Jose, CA, USA). Thus, the cell types considered positive in IF staining were validated by H&E staining.

## 3. Results

### 3.1. Immunochromatography (IC) Test Results

Initially, 50 dogs clinically suspected of CDV infection were screened using an immunochromatographic (IC) assay, yielding 22 positive (Figure 1A) and 28 negative (Figure 1B) results. To ensure sample quality, two IC-positive cases were excluded due to severe tissue autolysis, whereas three IC-negative dogs exhibiting distinct clinical signs (such as dyspnea, oculonasal discharges, and myoclonic tics) were retained for detailed investigation. Consequently, a cohort of 23 prospective cases (20 IC-positive and 3 IC-negative) was evaluated alongside 11 archived cases, comprising the final study material of 34 dogs. Among the three prospective cases that yielded negative results via the IC method, subsequent IHC staining confirmed that two were entirely CDV-negative across all evaluated organs. In contrast, the remaining discordant case demonstrated widespread CDV-positive immunoreactivity within the epithelial cells of the renal pelvis and urinary bladder, the pancreas, lymphoid tissues, and throughout the nervous system. Corroborating the IHC findings, RT-PCR analysis also confirmed that two of these three dogs were CDV-negative, while the third was positive. Notably, in this false-negative IC case, the density of CDV-infected cells within the lymphoid organs and tissues was markedly lower compared to the IC-positive cases, suggesting that a low antigen load may have fallen below the detection threshold of the rapid assay. When evaluating the diagnostic performance of the IC assay against the gold-standard reference methods (IHC and RT-PCR) strictly within this prospective cohort, the Overall Percent Agreement was determined to be 95.65% (22/23 concordant cases) (Table 2), as archived cases were inherently excluded from the validation baseline.

### 3.2. RT-PCR Results

Gel images of the 34 tested samples are presented (Figure 1C).

Although some low-contrast amplicons were observed in the RT-PCR analyses, bands of appropriate size were detected in all samples. These low-contrast amplicons were later confirmed using a separate RT-PCR. According to the RT-PCR results, CDV nucleic acid (RNA) was detected in 32 of the 34 samples, and these samples were considered positive. When RT-PCR and IHC results for CDV were compared, a complete correlation was observed. In one case that was negative by the IC method but immunopositive for CDV by IHC, the RT-PCR result was also found to be positive (Table 2).

### 3.3. Clinical Findings

Since the vast majority of cases in the study were strays or shelter-rescued animals, the ability to maintain complete, systematic clinical records was limited; consequently, the data had to rely primarily on medical history and findings from a general physical examination. The most commonly reported clinical findings included respiratory symptoms (cough, dyspnea, nasal/ocular discharge) and neurological signs (convulsions, tics) (Table 2). Respiratory system findings were reported in 25 of 34 cases (73.5%); no respiratory problems were observed in only one case, and an assessment could not be made in 8 cases. Neurological symptoms were present in 19 cases (55.9%), and it was determined that these cases generally involved dogs older than three months. Mucosal discharge (from the nose and eyes) was recorded in 29 cases (85.3%), was not observed in one case, and data could not be recorded for four cases. Upon evaluation of the clinical presentations, it was observed that despite the absence of overt neurological symptoms in the orphaned or early-separated puppies reared by surrogate nursing, immunohistochemical analysis revealed distinct CDV localization within the central nervous system.

### 3.4. Macroscopic Findings

In the dogs included in the study, nasal and ocular discharge ranging from serous to purulent was observed. On external examination, the animals were generally cachectic and dehydrated, and hyperkeratosis of the footpads was noted in some cases. In addition, enamel loss or dental caries was observed, particularly in the teeth of adult animals. In most cases, the lungs were not collapsed; instead, widespread hyperemia, edema, and serous fluid with costal impressions were observed. While the predominant pathological pattern was interstitial pneumonia, dark red hepatized areas and bronchopneumonia were also noted in most cases. In two cases, fibrinous bronchopneumonia and pleural adhesions were identified, while in one case, widespread abscess formation was observed in the lungs, mediastinal lymph nodes, and thymus.

One of the most striking findings was the presence of severe atrophy and hyperemia in the thymus observed in the vast majority of cases. The atrophy was so severe that, in some cases, the thymus had shrunk to the point where it could no longer be distinguished from the surrounding tissues. While the mediastinal and tracheobronchial lymph nodes were generally macroscopically unremarkable, enlargement and hyperemia were noted in some cases. In the spleen, a rough serosal surface and areas of focal hemorrhage were observed, accompanied by mild splenomegaly.

In the gastrointestinal tract, mild catarrhal gastritis in the stomach and a pattern of enteritis ranging from catarrhal to hemorrhagic in nature, extending throughout the small intestine and being more severe than in the stomach, were noted. In some dogs, infestations with ascarids and tapeworms were observed in the duodenum and jejunum. Ascarid infestations were generally found in young animals, while tapeworm infestations were observed in adults. The mesenteric lymph nodes were mostly found to retain their normal structure.

The urinary system was generally within normal limits; only in some cases were minimal areas of hyperemia observed in the urinary bladder mucosa. No pathological lesions were observed in the other organs, and they retained their normal anatomical structures.

### 3.5. Microscopic Findings

#### 3.5.1. Hematoxylin-Eosin Staining Results

Interstitial pneumonia and, in some cases (Figure 2A), purulent bronchopneumonia (Figure 2B) were observed in the lungs of dogs infected with CDV. In addition to these findings, an increase in the number of macrophages and type II pneumocyte hyperplasia was also observed. In some cases, syncytial giant cell formations were also observed (Figure 2C). In CDV-infected cases, eosinophilic intranuclear and intracytoplasmic inclusion bodies were observed in type II pneumocytes and in the mucosal epithelia of the bronchi and bronchioles (Figure 2D). Additionally, inclusion bodies were detected in alveolar macrophages and syncytial giant cells.

In the lymph nodes of the respiratory system (mediastinal and tracheobronchial lymph nodes), lymphocytolysis and neutrophil infiltration were noted, particularly in cases involving necrosis in the germinal centers of the follicles and purulent bronchopneumonia. Inclusion bodies were also observed in lymphocytes located primarily in the germinal centers of the follicles and in sinusoidal macrophages (Figure 2E).

A decrease in the number of thymocytes was observed in both the cortex and medulla of the thymus. It was determined that cell loss was quite significant in the cortex region, with the thickness of the cortex even decreasing to as little as 1–3 cells, and in some cases, the distinction between the cortex and medulla could not even be made; additionally, necrosis and intranuclear inclusion bodies were observed in thymocytes (Figure 2G).

In the liver, the histological structure was generally intact, but localized activation of Kupffer cells was observed. In two cases, multifocal areas of necrosis and neutrophil infiltration were noted (Figure 2H).

In the gastric mucosal epithelium of the cases included in the study, desquamation was observed, along with hyperemia and lymphocyte infiltration in the lamina propria, particularly in the submucosal layers. Inclusion bodies were also observed in the parietal cells and surface epithelial cells of the gastric gland epithelium (Figure 2I). Similar findings were noted in the duodenum, jejunum (Figure 2J), and ileum. Additionally, lymphocytolysis in Peyer’s patches and intranuclear eosinophilic inclusions in lymphocytes were noted in the ileum (Figure 2K).

In mesenteric lymph nodes, cases with intense CDV positivity have shown lymphocytolysis, vascular hyperemia, intranuclear inclusion bodies in lymphocytes within the follicular germinal center (less frequently in the perifollicular areas), and inclusions in perifollicular macrophages. In some cases, purulent lymphadenitis characterized by intense neutrophil infiltration and areas of necrosis was observed (Figure 2L).

In the pancreas of these cases, only small multifocal areas of necrosis (Figure 2M) were found in the exocrine pancreas, while the endocrine pancreas exhibited a normal histological structure.

In the spleens of CDV-positive cases, lymphocytolysis, necrosis, and, in some cases, neutrophilic leukocyte infiltration were noted. Necrosis was predominantly located in the germinal centers of lymphoid follicles. As in other lymphoid organs, eosinophilic intranuclear inclusions were detected in lymphocytes (Figure 2N). It was observed that the number of inclusions varied in direct proportion to the severity of necrosis. While inclusion bodies were present in lymphocytes, particularly in the germinal centers of the follicles, inclusion bodies were observed in sinusoidal macrophages in the peri- and para-follicular regions.

Examinations of the urinary system organs assessed the kidneys and the urinary bladder. The kidneys generally retained their normal histological structure; however, interstitial nephritis was observed in some cases, and purulent nephritis with clusters of neutrophils in the tubular lumens was noted in others. Hyaline casts were also observed in cases of nephritis. The most prominent lesions associated with CDV were found in the renal pelvis. Hydropic degeneration, as well as intranuclear and intracytoplasmic inclusion bodies, were observed in the renal pelvis epithelium. It was determined that the inclusions were particularly present in areas with hydropic degeneration. In the urinary bladder, lesions similar to those in the renal pelvis were observed (Figure 2O). Additionally, lymphocytic infiltrations were observed in the lamina propria of the regions where inclusions and hydropic degeneration were present.

Hyperkeratosis was observed in the footpads and planum nasale; orthokeratotic and parakeratotic hyperkeratosis predominated, particularly in areas of the epidermis with focal hyperplasia. In the dermis, lymphocytic infiltrates were noted in some areas. In the epidermis, particularly in the stratum spinosum and stratum granulosum layers, focal hydropic degeneration was observed, along with intranuclear and intracytoplasmic eosinophilic inclusion bodies in these cells (Figure 2P).

Various sections of the central nervous system, including the brain, cerebellum, brainstem, and spinal cord, were examined. Areas of neuronophagia and focal necrosis were observed in the brain tissue of the cases. Glial cell proliferation was noted around the third ventricle and lateral ventricles; additionally, hyperemia in the choroid plexuses was observed in some cases. In cases where severe demyelination was present around the fourth ventricle (Figure 2Q), demyelination was also found to be prominent in the lateral and third ventricle areas. Eosinophilic inclusion bodies were observed in the ependymal cells covering the ventricles and in the astrocytes in areas of intense demyelination (Figure 2R). In two cases, parasitic cysts interpreted as *Toxoplasma* pseudocysts were identified; however, apart from mild gliosis, no other inflammatory reactions were observed in these areas.

The most prominent lesions in the cerebellum were observed to be demyelination around the fourth ventricle and in the cerebellar peduncle, along with inclusion bodies in the astrocytes and ependymal cells in these regions (Figure 2S). Hydropic degeneration was observed in ependymal cells, and inclusion bodies were found to be more concentrated in these regions. In addition to these findings, gliosis, perivascular mononuclear cell infiltration (Figure 2T), and neuronophagia were detected.

In the brainstem, near the choroid plexus, hydropic degeneration and necrosis in ependymal cells, along with intranuclear inclusion bodies in astrocytes in these regions, were identified. Focal areas of necrosis and gliosis were observed in the parenchyma (Figure 2U), and mild demyelinated areas were noted in the tissue near the choroid plexus. Compared to the cerebellum, demyelination was found to be milder, while perivascular cellular infiltrations were at a similar level. Unlike the brain and cerebellum, necrosis and intracytoplasmic inclusion bodies were detected in motor neurons.

Intracellular and intranuclear inclusion bodies have been observed in motor neurons and intranuclear inclusion bodies in astrocytes (Figure 2V) in association with demyelination of the spinal cord. It has been determined that the inclusions in astrocytes generally form in the substantia grisea and regions adjacent to the central canal. In some cases, inclusion bodies were also detected in the ependymal cells of the central canal (Figure 2W), and additionally, necrosis and neuronophagia were observed in motor neurons (Figure 2X).

In control dogs, no lesions related to CDV were found in any tissues or organs, and the tissues generally exhibited a normal histological structure.

#### 3.5.2. CDV Immunohistochemical Findings

CDV-positive reactions in tissues and organs are shown in Table 3.

Regarding lung tissues, CDV positivity was detected in 27 of the 34 dogs included in the study, while the lungs were negative in seven cases. In five of the cases with negative lung findings, positive reactions were noted in other organs. The remaining two cases were completely CDV-negative. CDV-positive reactions in the lungs were evident primarily in bronchial and bronchiolar epithelial cells, as well as in type II pneumocytes (Figure 3A) and alveolar macrophages (Figure 3B). In areas with interstitial pneumonia, bronchial and bronchiolar epithelial cells were predominantly positive, whereas in cases with purulent bronchopneumonia, a decrease in CDV-positive reactions in bronchial and bronchiolar epithelial cells was observed. Furthermore, inclusion bodies were occasionally identified in CDV-positive cells, and both these cells and the inclusion bodies were found to be CDV-positive.

The mediastinal and tracheobronchial lymph nodes from 23 cases were examined immunohistochemically, and 20 of them were found to be CDV-positive. Although the two negative cases were completely negative, in one case, CDV positivity was detected in the CNS, a limited number of bronchiolar mucosal epithelial cells and type II pneumocytes in the lungs, renal pelvis epithelium, and squamous cells in the basal lamina, while the positive reactions in the lymph nodes were generally observed in lymphocytes within the germinal centers of the follicles; in 15 of the 20 positive cases (Figure 3C), positivity was also observed in the perifollicular area in addition to the germinal centers, however, the reaction in the perifollicular area was noted to be less intense compared to that in the germinal center. Additionally, CDV-positive reactions were observed in macrophages; it was determined that inclusion bodies were present in some of the positive lymphocytes and that these inclusions also exhibited a positive reaction (Figure 3D).

Thymic tissue from 20 cases was evaluated; CDV positivity was detected in 16 of these cases (Figure 3E), while four cases were found to be CDV-negative. In the positive cases, the immunohistochemical reaction was observed in both the cortex and the medulla; however, the reaction in the medullary region was noted to be more intense compared to that in the cortex.

Hepatic tissue was assessed in 32 cases, with 18 demonstrating positive CDV immunoreactivity of varying intensities. The immunohistochemical reaction was found to be particularly concentrated in Kupffer cells, and positivity was also observed in a small number of bile duct epithelial cells (Figure 3F). It was determined that in all dogs with CDV-positive livers, the organs of the digestive system were also positive.

In the study, the stomachs of 28 out of 34 cases were examined, and CDV positivity was detected in 23 of them. Immunohistochemical reactions were observed at the levels of the *Lamina Epithelialis* and *Lamina Propria*. Positivity was particularly noted in the surface mucosal epithelium (Figure 3G), glandular epithelium, and lymphocytes and macrophages in the lamina propria. Intranuclear inclusion bodies were detected in positive cells, and CDV reactivity was identified within these inclusions.

The duodenum was evaluated in 32 cases, showing CDV positivity in 18 (Figure 3H). Immunopositive reactions were found in villus and crypt epithelial cells, as well as in infiltrating lymphocytes and macrophages in the lamina propria. The positivity in crypt epithelial cells was more intense compared to that in villous epithelium, and strong staining was also noted in inflammatory cells within the villous lamina propria. Brunner’s gland epithelia were predominantly infected, and numerous CDV-positive intranuclear inclusion bodies were detected in these cells.

Jejunal examination in 33 dogs revealed CDV positivity in 19 cases (Figure 3I). The positive reaction was observed in villus and crypt epithelial cells as well as in inflammatory cells within the lamina propria. It was determined that the reactions were more intense in infiltrating lymphocytes and macrophages compared to those in epithelial cells.

The ileum of 32 out of 34 cases was evaluated, and CDV positivity was detected in 22 of them (Figure 3J). Positive reactions were detected in villus and crypt epithelial cells, inflammatory infiltrative cells, and Peyer’s patches. In one case, the villi and crypts were positive while the Peyer’s patches were negative; in four cases, the villi and crypts were negative while the Peyer’s patches were positive. It was noted that the CDV reaction in lymphocytes within the Peyer’s patches was quite intense, while positivity in the crypt epithelium was particularly concentrated in regions adjacent to the Peyer’s patches.

Examination of mesenteric lymph nodes from 21 cases revealed CDV positivity in 16 (Figure 3K). While a reaction was observed in lymphoid cells within the germinal centers of the follicles in all positive cases, positive cells were also observed in the perifollicular area in 12 cases. Similar to other lymphoid organs, the positivity was found to be particularly concentrated in the germinal centers, while that in the lymphocytes of the perifollicular region was quite low. Positive reactions were frequently observed in macrophages in the perifollicular areas, and intranuclear inclusion bodies, along with CDV positivity within these inclusions, were detected in germinal center lymphocytes.

In the study, the pancreas was examined in 32 of 34 cases, and CDV positivity was detected in 20 of these (Figure 3L). The immunohistochemical reaction was observed primarily in exocrine pancreatic cells, while no CDV-positive reaction was found in the endocrine pancreas.

Splenic tissue from 30 dogs demonstrated CDV positivity in 21 cases (Figure 3M). It was observed that, in addition to lymphocytes, macrophages, particularly those located in the perifollicular regions, also exhibited a positive reaction to CDV. In positive cases, intense immunoreactivity was observed in lymphocytes within the follicular germinal centers, while in 14 cases, a small number of lymphocytes in the perifollicular area were also found to be positive. Additionally, it was determined that the inclusion bodies present in the lymphocytes of the follicular germinal centers were also CDV-positive.

The kidney tissues of the 34 dogs included in the study were evaluated immunohistochemically, and CDV positivity was detected in 24 cases (Figure 3N). In all positive cases, the reaction was particularly concentrated in the renal pelvis epithelium. In two cases, positivity was also observed in a very small number of renal tubular epithelial cells, and it was noted that these cells were localized in the medulla around the renal pelvis. In one case, a CDV-positive reaction was observed in glomerular vascular endothelium and mesangial cells. In cases where interstitial nephritis was identified on Hematoxylin-Eosin staining, no CDV-positive reaction was observed in the infiltrating lymphocytes; based on this finding, it was concluded that the interstitial nephritis was likely due to a secondary infection or co-infection.

The urinary bladder was examined in 28 cases, and a CDV-positive reaction was detected in 19 of them (Figure 3O). It was determined that the positivity was present in the urothelial cells of the mucosa and in the few inflammatory cells infiltrating the lamina propria. It was noted that in cases where the urinary bladder was positive, the renal pelvic epithelial cells were also generally positive.

In the study, tissue from the footpads was examined in 25 of 34 cases, and a CDV-positive reaction was detected in 15 of them. It was determined that the positivity was present in the stratified squamous epithelial cells of the epidermis and in the sweat gland epithelial cells of the dermis. It was noted that the reaction was intense in the stratum spinosum, stratum granulosum, and stratum corneum of the epidermal layers, while it was weaker in the stratum germinativum.

Concurrently, the planum nasale from 26 cases was evaluated, showing CDV positivity in 15 (Figure 3P). In the planum nasale as well, positive reactions were observed in the epidermal squamous cells and the sweat gland epithelial cells in the dermis. Similar to the footpads, it was found that the reaction in the nasal epidermis was intense in the stratum spinosum, stratum granulosum, and stratum corneum, while it was weaker in the basal layer.

In the study, the brains of 33 out of 34 cases were examined, and CDV immunopositivity was detected in 25 of them. Although the severity of the reaction varied among cases, it was generally concentrated in the lateral white matter and around the third ventricle (Figure 3Q). Positive staining was observed particularly in astrocytes in demyelinated areas and in ependymal cells lining the ventricular walls. Positivity was also observed in astrocytes within the brain parenchyma outside the ventricles; the reaction was found to be prominent around blood vessels, and vascular endothelium in these regions was also positive. In a small number of cases, intense positivity was detected in astrocytes of the substantia grisea, as well as in astrocytes of the cornu ammonis. While the reaction in the cornu ammonis was intense in astrocytes, neurons were generally negative; it was noted that positivity was present in only a small number of neurons in two cases (Figure 3R). When the brain, cerebellum, and brainstem were evaluated together, it was observed that in some dogs, the cerebellum and brainstem were positive while the brain was negative, and that immune reactions were generally more intense in the cerebellum and brainstem than in the brain.

The cerebellum was examined in all 34 cases, and CDV positivity was detected in 27 of them. It was determined that the positive reactions were concentrated around the fourth ventricle and that demyelination was more pronounced in these regions (Figure 3S). Immune reactions in the astrocytes of the white matter around the fourth ventricle were found to be more intense compared to those in other brain regions (Figure 3T) and in severe cases, intense positivity was also observed in the choroid plexuses and ependymal cells (Figure 3U). Intranuclear inclusion bodies were detected in astrocytes, and CDV positivity was identified within these inclusions (Figure 3T). In some orphaned or early-separated puppies reared by surrogate nursing that did not exhibit clinical neurological symptoms, positive immunoreactions were observed within the choroid plexuses, while the cerebellum remained negative.

The brainstem was examined in all 34 cases, and CDV positivity was detected in 25 of them. In 10 of the positive cases, the reaction was detected only in astrocytes; in 11 cases, it was detected in both astrocytes and motor neurons (Figure 3V); and in 4 cases, it was detected only in motor neurons. While the immunoreactivity in astrocytes was similar to that in the brain and cerebellum, the intensity of positivity in motor neurons was notably higher. It was noted that in some motor neurons, the intense CDV staining made it difficult to distinguish between the cell nucleus and cytoplasm; additionally, mild or weak granular staining was observed in some neurons.

In the study, the spinal cord was examined in 23 of 34 cases, and CDV positivity was detected in 17 of them (Figure 3W). It was determined that positive reactions occurred in astrocytes in both the substantia grisea and the substantia alba, as well as in some motor neurons in the substantia grisea and in the ependymal cells of the central canal (Figure 3X). In some cases, intense reactions were observed in astrocytes, while in others, weak granular positivity was observed in motor neurons without any reaction in the astrocytes. The intensity of CDV positivity in motor neurons varied among cases; in cases where the immune reaction was severe, the ependymal cells of the central canal were generally intensely positive.

#### 3.5.3. CD150 Immunohistochemical Findings

The distribution and intensity of CD150 expression across various tissues and organs of dogs with distemper are summarized in Table 4. Immunohistochemical analysis revealed a positive immunoreaction generally localized within the cytoplasm of lymphocytes and sinusoidal macrophages in the thymus, spleen, and lymph nodes (Figure 4A–C). Furthermore, CD150 positivity was detected in alveolar macrophages in the lungs (Figure 4D), in inflammatory infiltrating cells in the gastrointestinal tract (Figure 4E), in lymphocytes and macrophages in Peyer’s patches (Figure 4F), and in the cytoplasm of Kupffer cells in the liver (Figure 4G). In the kidney, an immune reaction was detected in a small number of lymphocytes in the renal pelvises; in the urinary bladder, in infiltrating lymphocytes within the lamina propria; in inflammatory infiltrates of the nasal mucosa and nasal septum; and in the central nervous system, in a very small number of lymphocytes and microglia (Figure 4H). In addition to the infiltrating lymphocytes and macrophages in these tissues and organs, a CD150-positive immunoreaction was also observed in monocytes located within the lumen of blood vessels throughout all tissues and organs (Figure 4I).

#### 3.5.4. Nectin-4 Immunohistochemical Findings

The IHC staining results for Nectin-4 in the examined tissues and organs are given in Table 5. Immunohistochemical staining for Nectin-4 in the examined tissues and organs revealed that many epithelial cells were positive. In the lungs, Nectin-4 positivity was detected in the bronchial and bronchiolar mucosal epithelium and bronchial glands (Figure 5A), as well as in endothelial cells of the intima layer of some blood vessels and in neurons at peripheral nerve endings (Figure 5B). In the stomach, positive reactions were observed in the mucosal surface epithelium, glandular epithelium, and parietal cells (Figure 5C); in the intestines, in villus and crypt epithelial cells (Figure 5D). It was noted that this reaction was more intense in the stomach and jejunum compared to those in other parts of the digestive system, and among intestinal segments, the highest intensity of positivity was observed in the jejunum. In the liver, positivity was observed in bile duct epithelial cells, while nonspecific staining was noted in hepatocytes. In the kidney, Nectin-4 positivity was detected in some tubular epithelial cells and in the renal pelvis epithelium (Figure 5E,F); in the urinary bladder, it was detected in all mucosal epithelial cells (Figure 5G). In the footpads and planum nasale, all epidermal layer cells were positive; however, the reaction in the basal layer was weaker compared to other layers (Figure 5H), and positivity was also detected in the sweat gland epithelial cells of the dermis (Figure 5I). In the central nervous system, Nectin-4 positivity was observed in ependymal cells of the brain, cerebellum, brainstem, and spinal cord; in the choroid plexus epithelium (Figure 5J); and in the ependymal cells of the central canal of the spinal cord, along with a small number of neurons; it was determined that the reaction was more intense in motor neurons (Figure 5K) of the substantia grisea of the spinal cord compared to other regions of the nervous system. While weak Nectin-4 positivity was observed in the endothelial cells of the intima layer of some blood vessels in all organs (Figure 5L), the heart, spleen, thymus, and lymph nodes were negative, and nonspecific staining was observed throughout the pancreas.

#### 3.5.5. GFAP Immunohistochemical Findings

In immunohistochemical staining for GFAP, a positive reaction was observed in the brain, cerebellum, and brainstem, particularly in the white matter, and in the spinal cord, specifically in the gray matter. GFAP-positive astrocyte processes typically exhibit a star-like appearance and are localized between blood vessels and neurons. It was found that GFAP positivity is more intense in the brain’s white matter and weaker in the gray matter (Figure 6A). In the brain, the positive reaction was found to be particularly intense in the granular layer, while in the pyramidal layer, only astrocytic extensions showed a positive reaction. In the cerebellum (Figure 6B), GFAP-positive reactions were observed in cells of the molecular and granular layers; it was noted that positive cells were also present in the substantia alba, and that the density there was higher than in the substantia grisea. In the brainstem, GFAP immunoreactivity was observed to form in the substantia alba, similar to the brain and cerebellum. In the spinal cord, however, it was determined that GFAP-positive cells were predominantly located in the substantia grisea (Figure 6C,D).

#### 3.5.6. Immunofluorescence Staining Results

##### Triple Immunofluorescence

In this study, triple immunofluorescence (IF) staining was performed to simultaneously evaluate the localization of CDV antigen alongside the host receptors CD150 and Nectin-4. However, the heart was CDV-negative, Nectin-4 staining in the pancreas was nonspecific, and there was excessive background staining in hepatocytes in the liver; therefore, these organs were not evaluated using the triple IF method.

In triple immunofluorescent staining of the lungs, CDV-positive reactions were detected in bronchial and bronchiolar epithelial cells, bronchial glands, type II pneumocytes, and alveolar macrophages (Figure 7(Aa)). CDV positivity was also observed in inflammatory infiltrating cells in lungs with severe bronchopneumonia. Alveolar macrophages were found to be CD150-positive; while a CD150-positive reaction was also observed in lymphocytes within areas of inflammatory infiltration, this reaction was determined to be weaker compared to that in alveolar macrophages (Figure 7(Ab)). Bronchial and bronchiolar mucosal epithelial cells, bronchial gland epithelial cells, and type II pneumocytes were found to be Nectin-4 positive (Figure 7(Ac)); this reaction was intense in bronchial and bronchiolar epithelial cells, while it was weak in bronchial glands and type II pneumocytes. Upon combining the triple IF images, it was found that macrophages and infiltrating lymphocytes were CD150- and CDV-positive, whereas bronchial and bronchiolar epithelial cells, bronchial gland epithelia, and type II pneumocytes were positive for both CDV and Nectin-4 (Figure 7(Ad)). By overlaying the IF and H&E images of the same sections, it was confirmed that CDV infects these cell types in the lungs based on their cellular morphology (Figure 7(Ae,Af)).

In triple IF staining of mediastinal and tracheobronchial lymph nodes, CDV-positive lymphocytes were observed particularly in the germinal centers of lymphoid follicles, and CDV positivity was also detected in a small number of macrophages in the same areas. Although the number of CDV-positive cells was low in perifollicular lymphocytes and macrophages, an increase in the number of positive cells was observed in perivascular areas (Figure 7(Ba)). Similarly, in CD150 IF staining, a high number of positive cells was observed in follicular germinal centers, whereas the number was low in perifollicular regions (Figure 7(Bb)). No Nectin-4-positive reaction was observed in lymph nodes outside of vascular endothelium (Figure 7(Bc)). Overlaying the IF images revealed that lymphocytes and macrophages in lymph nodes were CDV and CD150 positive (Figure 7(Bd)). By combining IF and H&E images of CDV-positive areas, it was confirmed that the immunoreactive reaction was particularly present in lymphocytes and macrophages, and that some lymphocytes contained intranuclear eosinophilic inclusion bodies (Figure 7(Be,Bf)).

In triple immunofluorescent staining performed on the thymus, thymocytes were found to be widely CDV-positive (Figure 7(Ca)). While thymocytes also showed an immunopositive reaction in CD150 staining (Figure 7(Cb)), only mild positivity was observed in vascular endothelium in Nectin-4 staining (Figure 7(Cc)). Overlaying the staining results revealed that thymocytes were positive for both CD150 and CDV; unlike in other lymphoid organs, nearly all CD150-positive thymic lymphocytes were also found to be positive for CDV (Figure 7(Cd)). By combining IF and H&E images, it was confirmed that CDV-positive cells were thymic lymphocytes, and it was determined that positivity was particularly present in thymocytes located in the medullary portion of the thymus (Figure 7(Ce,Cf)).

The findings obtained using the H&E mounting method combined with triple immunofluorescent staining of the stomach, duodenum, jejunum, ileum, and mesenteric lymph nodes are as follows: CDV positivity was detected in macrophages and lymphocytes in the lamina epithelialis and lamina propria of the stomach (Figure 7(Da)); CD150 was observed, particularly in macrophages at the junction of the tunica muscularis and tunica mucosa, where it was intense and present in areas with inflammatory infiltration (Figure 7(Db)), while Nectin-4 was positive in surface epithelial cells (Figure 7(Dc)). In the triple IF overlay, mucosal epithelial cells were positive for Nectin-4 and CDV, while lymphocytes and macrophages in the lamina propria were positive for CD150 and CDV (Figure 7(Dd)); in the H&E overlay, it was confirmed that mucosal surface and glandular epithelial cells, parietal cells, and macrophages and lymphocytes in the lamina propria were infected with CDV (Figure 7(De,Df)). In the duodenum, CDV positivity was observed in areas with intense lymphocytic infiltration, particularly in cases of enteritis (Figure 7(Ea)); CD150 positivity was detected in lymphocytes (Figure 7(Eb)), and Nectin-4 positivity was detected in villus and crypt epithelial cells (Figure 7(Ec)). In immunofluorescence (IF) co-staining, macrophages and lymphocytes were positive for CD150 and CDV, while villus and crypt epithelial cells were positive for Nectin-4 and CDV (Figure 7(Ed)); H&E staining confirmed that CDV infects these cell types (Figure 7(Ee,Ef)). Villus and crypt epithelial cells in the jejunum, as well as lymphocytes and macrophages in the lamina propria, were CDV-positive (Figure 7(Fa)); CD150 was positive in a small number of inflammatory cells infiltrating the lamina propria (Figure 7(Fb)), while Nectin-4 was positive in villus and crypt epithelia (Figure 7(Fc)); In immunofluorescence (IF) co-staining, lymphocytes/macrophages were positive for CD150 and CDV, while the epithelia were positive for Nectin-4 and CDV (Figure 7(Fd)); H&E staining confirmed that these cells were infected with CDV (Figure 7(Fe,Ff)). In the ileum, villus and crypt epithelia, inflammatory cells in the villus lamina propria, and lymphocytes in Peyer’s patches were CDV-positive, with a more intense reaction particularly in crypt epithelia near Peyer’s patches (Figure 7(Ga)). CD150 was positive in Peyer’s patch lymphocytes and infiltrating inflammatory cells (Figure 7(Gb)), while Nectin-4 was positive in villus and crypt epithelial cells (Figure 7(Gc)); In immunofluorescence (IF) staining, Peyer’s patches and inflammatory cells were positive for CD150 and CDV, while epithelial cells were positive for Nectin-4 and CDV (Figure 7(Gd)); in H&E staining, it was confirmed that CDV-positive cells include villus/crypt epithelium, infiltrating lymphocytes/macrophages, and Peyer’s patch lymphocytes (Figure 7(Ge,Gf)). In mesenteric lymph nodes, CDV was positive in lymphocytes within follicular germinal centers; in the perifollicular area, macrophages and a small number of lymphocytes were CDV-positive (Figure 7(Ha)); CD150 was also positive in germinal center lymphocytes (Figure 7(Hb)), while Nectin-4 was negative (Figure 7(Hc)). In immunofluorescence (IF) staining, germinal center lymphocytes and macrophages were CD150- and CDV-positive, while the reaction was weaker in the perifollicular area (Figure 7(Hd)); H&E staining confirmed that the CDV-positive cells were morphologically consistent with lymphocytes and macrophages (Figure 7(He,Hf)).

Triple immunofluorescence staining of the spleen revealed strong immunopositivity for CDV in lymphocytes located in the germinal centers of lymph follicles, similar to other lymphoid tissues, while a small number of cells in the perifollicular areas showed a positive CDV reaction (Figure 8(Aa)). CD150 staining was also observed to occur generally in the follicular germinal centers, with a weaker immune reaction in cells in the perifollicular area (Figure 8(Ab)). The spleen was determined to be negative for Nectin-4 (Figure 8(Ac)). Overlaying of the IF images revealed that lymphocytes and macrophages in the follicular centers were positive for CDV and CD150 (Figure 8(Ad)). Combining CDV-positive areas with H&E-stained sections confirmed that the CDV-positive cells were lymphocytes and macrophages based on their morphology (Figure 8(Ae,Af)).

Triple IF staining of the kidneys revealed a positive CDV reaction, particularly in the renal pelvis epithelia (Figure 8(Ba)). CD150 positivity occurred in the intertubular areas of the renal cortex and medullary region, as well as in the inflammatory infiltrative cells of the renal pelvis; areas without inflammatory infiltration were negative (Figure 8(Bb)). Nectin-4 staining showed positivity in the tubular epithelial cells of the renal cortex and medullary regions, and in the renal pelvis epithelia (Figure 8(Bc)). Overlaying of the IF images revealed Nectin-4 and CDV positivity in the renal pelvis epithelia, and CD150- and CDV-positivity in the inflammatory infiltrative cells; areas without inflammatory infiltration were negative for these markers (Figure 8(Bd)). Combining CDV-positive areas with H&E images revealed that CDV-positive reactions occurred in the renal pelvis epithelia and inflammatory infiltrative cells (Figure 8(Be,Bf)).

In the urinary bladder CDV IF staining, positive reactions were found in inflammatory infiltrative cells of the mucosal epithelium and lamina propria (Figure 8(Ca)). CD150 positive reaction was observed in the lamina propria, especially in lymphocytes and macrophages, while negative staining was seen in areas without infiltration (Figure 8(Cb)). Nectin-4 staining revealed that all bladder mucosal epithelia and rarely vascular endothelia were positive (Figure 8(Cc)). Overlaying of IF images showed that mucosal epithelia were positive for both CDV and Nectin-4 (Figure 8(Cd)). Combining CDV-positive areas with H&E images confirmed that mucosal epithelial cells were CDV-positive; furthermore, intracytoplasmic inclusion bodies were observed in CDV-positive cells in some areas (Figure 8(Ce,Cf)).

Triple IF staining of the footpads and planum nasale tissues revealed CDV-positive reactions in squamous cells and glandular epithelia in the epidermal layer (Figure 8(Da)). CD150 positivity was observed in inflammatory infiltrative cells in the dermis, while the epidermis was determined to be CD150-negative (Figure 8(Db)). Nectin-4 staining showed weak positivity in epidermal cells and glandular epithelia (Figure 8(Dc)). Overlaying of the IF images revealed that epidermal layer cells and glandular epithelia were positive for both Nectin-4 and CDV (Figure 8(Dd)). Combining CDV-positive areas with H&E images confirmed CDV positivity in keratinocytes and sweat gland epithelia in the stratum spinosum, stratum granulosum, and stratum basale layers of the epidermis (Figure 8(De,Df)).

Triple IF staining of brain, cerebellum, brainstem, and spinal cord revealed CDV-positive reactions in astrocytes, ependymal cells, choroid plexus epithelia, and motor neurons, based on cell morphology and localization (Figure 8(Ea)). In the brain, CD150 positivity was observed in a small number of cells (possibly infiltrative lymphocytes), but these cells were CDV-negative (Figure 8(Eb)). Nectin-4 was positive in choroid plexus epithelia, ependymal cells, and rarely in some neurons (as a thin line in the plasma membrane and weak cytoplasmic staining) (Figure 8(Ec)). IF overlay showed that choroid plexus epithelia and ependymal cells were positive for both Nectin-4 and CDV, while astrocytes, where CDV was most abundant, were negative for both Nectin-4 and CD150 (Figure 8(Ed)). H&E overlay confirmed CDV positivity in astrocytes, ependymal cells, and choroid plexus epithelia (Figure 8(Ee,Ef)). Similarly, CDV positivity was observed in astrocytes, ependymal cells, and the epithelia of the 4th ventricle choroid plexus; the CDV reaction was generally predominant in astrocytes surrounding the 4th ventricle (Figure 8(Fa)). CD150 was positive in very few cells (Figure 8(Fb)), while Nectin-4 was positive in choroid plexus epithelia, ependymal cells, and a small number of neurons (peripheral cytoplasmic) (Figure 8(Fc)). In IF overlay, the epithelia of the 4th ventricle choroid plexus, ependymal cells, and a small number of neurons were positive for CDV and Nectin-4; inflammatory infiltrative cells were positive for CDV and CD150; and astrocytes were negative for Nectin-4 and CD150 (Figure 8(Fd)). H&E overlay confirmed CDV positivity in astrocytes, ependymal cells, and choroid plexus epithelia (Figure 8(Fe,Ff)). In the brainstem, CDV positivity was observed in astrocytes and motor neurons (Figure 8(Ga)); CD150 was positive in a small number of cells (Figure 8(Gb)), while Nectin-4 showed weak positivity as a thin line on the plasma membrane of neurons (Figure 8(Gc)). In IF overlay, motor neurons were positive for Nectin-4 and CDV, astrocytes were positive for CDV but negative for Nectin-4 and CD150 (Figure 8(Gd)); H&E overlay confirmed that astrocytes and motor neurons were CDV-positive (Figure 8(Ge,Gf)). In the spinal cord, CDV positivity was detected in astrocytes, motor neurons, and central canal epithelia (Figure 8(Ha)); CD150 was negative (Figure 8(Hb)), while Nectin-4 was positive in central canal ependymal cells and motor neurons (Figure 8(Hc)). In IF overlay, central canal ependymal and motor neurons were positive for Nectin-4 and CDV, astrocytes were CDV-positive but negative for Nectin-4 and CD150 (Figure 8(Hd)). H&E overlay showed that astrocytes and motor neurons were CDV-positive, and inclusion bodies were also observed in CDV-positive motor neurons and astrocytes (Figure 8(He,Hf)).

##### Double Immunofluorescence

In double immunofluorescence staining performed with GFAP and CDV markers in brain, cerebellum, brainstem, and spinal cord sections (DAPI was used for nucleus staining), astrocytes were found to be positive for both CDV and GFAP. In the brain, CDV-positive cells were observed to be more concentrated in the substantia alba (Figure 9A), and GFAP-positive astrocytes were also more abundant in this region (Figure 9B); combining CDV, GFAP, and DAPI images confirmed that CDV-positive cells were astrocytes (Figure 9D). Thus, it was confirmed that cells that were found to be CDV-positive but negative for Nectin-4 and CD150 receptors in previous IHC and triple IF methods were astrocytes. In the cerebellum (Figure 9E–H), GFAP-positive astrocytes were located in both the substantia grisea and alba layers, and CDV positivity occurred in the same regions; image combining determined that astrocytes were CDV-positive. Similarly, in the brainstem (Figure 9I–L) and spinal cord (Figure 9M–P), astrocytes were found to be positive for both CDV and GFAP; In triple IF, it was confirmed that cells that were CDV-positive but Nectin-4 and CD150-negative were indeed astrocytes.

## 4. Discussion

Canine distemper is a contagious viral infection commonly seen in carnivores worldwide and in Türkiye. The causative agent is the canine distemper virus, belonging to the *Morbillivirus* genus of the *Paramyxoviridae* family, which shows a close antigenic relationship with measles, Peste des petits ruminants, and rinderpest viruses. CDV causes a multisystemic infection; it forms lesions in numerous systems and organs, including mucosal epithelial cells in the respiratory, digestive, and urinary systems, as well as the lymphoreticular system, skin, and central nervous system.

Studies on canine distemper virus (CDV) receptors have revealed that viral receptors identified for the measles virus, with which it is antigenically closely related, are also used by CDV. Consistent with the tropism observed in other morbilliviruses, CDV and rinderpest virus also rely on the CD150 receptor for infecting lymphoid populations, mirroring the cellular entry process of the measles virus [8,17]. CDV utilizes the CD150 receptor for lymphocytic cell entry, a receptor that is widely expressed across diverse immune populations, including immature thymocytes, activated T and B lymphocytes, monocytes, tissue macrophages, and dendritic cells [18,19,20,21,22]. On the other hand, the Nectin-4 receptor has been identified as the key mediator for CDV entry into epithelial cells, similar to its role in human measles virus infection [9]. It has been shown that the Nectin-4 receptor is expressed in bronchial, gastric, and intestinal gland epithelial cells, urinary bladder pseudostratified epithelium, renal pelvis epithelium, tonsil epithelium, epidermal keratinocytes, and some central nervous system cells (neurons, ependymal cells, etc.), and that CDV uses this receptor to infect epithelial cells [23,24,25]. However, it is known that CD150 and Nectin-4 receptors are absent in astrocytes, which are the most frequently infected cells by CDV in the CNS [25]. Therefore, it is thought that CDV may use different pathways to infect astrocytes, although this has not yet been discovered [11,12,13].

Clinical, hematological, and macroscopic findings remain insufficient for a definitive diagnosis of CDV infections, necessitating the implementation of immunological or molecular methods. In this study, nasal and ocular swab samples from the 23 prospective suspected CDV cases were evaluated using a commercial immunochromatographic (IC) rapid diagnostic kit, yielding 20 positive and three negative results. Among the three IC-negative cases, two were confirmed as true negatives by both IHC and RT-PCR. Conversely, one case presented a discordant profile, testing negative by IC but positive by both IHC and RT-PCR. In the literature, similar diagnostic sensitivities have been reported between IC and PCR assays in mucosal and cerebrospinal fluid (CSF) samples [26], with some studies documenting a complete one-to-one overlap [27]. In contrast, other comparative analyses have demonstrated the superior sensitivity of RT-PCR [28]. In the present study, the rapid IC assay demonstrated a high Overall Percent Agreement (OPA) of 95.65% (22/23 concordant cases) strictly within the prospective cohort. This high level of agreement is largely attributed to the fact that the majority of these clinical cases were in the acute phase of infection. However, the single false-negative IC result occurred in a chronic case, driven by a low mucosal viral load in the nasal and ocular passages during the later stages of the disease. This biological limitation is further supported by our finding that IHC positivity in chronic cases was prominently higher in the central nervous system (CNS) compared to peripheral mucosal organs. Consequently, despite the high cohort-specific agreement rate of the rapid IC method, RT-PCR and tissue IHC techniques proved to be more sensitive and definitive for comprehensive diagnosis, which is consistent with established literature [28].

The RT-PCR technique is widely used in the diagnosis of CDV infection due to its rapid and reliable nature. In this study, organ mixtures prepared from the lungs, thymus, liver, stomach, intestines, mesenteric lymph nodes, kidneys, spleen, cerebellum, and footpads of 34 cases were evaluated using RT-PCR, and 32 cases were found to be CDV-positive. Immunohistochemistry also yielded positivity in all of the same cases, demonstrating the high sensitivity of both techniques. In an experimental study, viral nucleic acids were detected in ocular swabs starting from the first day of infection, in nasal irrigation and peripheral blood from the third day, and in urine from the fourth day. However, they remained undetectable in the cerebrospinal fluid (CSF) up to and including the 14th day, with a reported decrease in viral load across nasal, ocular, and urine samples during this period [29]. Findings indicate that the onset of demyelination typically takes place around the 21st day post-infection, a stage at which extraneural clinical signs tend to resolve [1]. In light of this literature data, it was observed in this study that the viral load in organs and secretions changes in different stages of the disease; that IC, RT-PCR, and IHC sensitivities are high in the acute phase, but the sensitivity of the IC method decreases in the chronic phase, while the RT-PCR and IHC methods maintain their sensitivity.

Canine distemper virus causes a multisystemic infection resulting in clinical symptoms affecting different systems, including high fever, malaise, respiratory distress, cough, serous to purulent nasal and tear discharge, diarrhea, vomiting, dehydration, hyperkeratosis of the nasal and footpads, and convulsions and tics [30,31,32,33,34]. Similarly, in this study, nasal and tear discharge, severe respiratory problems, and neurological symptoms were frequently observed in most cases. Of 34 cases, 19 showed neurological symptoms, and 17 of these were found to be CDV-positive by RT-PCR and IHC. Of the 15 cases without neurological symptoms, 14 demonstrated IHC positivity in the CNS; these cases were assessed as acute distemper infections in orphaned puppies reared by surrogate nursing. Literature reports that CNS infection begins in the choroid plexuses approximately 10 days after infection, with demyelination and neurological symptoms appearing approximately 21 days later [1,35,36]. Accordingly, the absence of neurological symptoms in the puppies in this study was attributed to the virus causing death in the acute phase before lesion formation in the brain tissue. Indeed, in these cases, while CDV positivity was observed in the choroid plexuses and ependymal cells surrounding the ventricle using IHC, limited reaction was noted in the brain and cerebellar parenchyma. Consistent with the acute stages of infection in young animals, puppies presumed to be colostrum-deficient frequently demonstrated faint CDV immunopositivity within the CNS, accompanied by little to no associated demyelination [37].

In this study, lesions consistent with the disease were observed under a light microscope in H&E-stained tissue sections taken from the respiratory, digestive, urinary, and central nervous system organs, as well as the planum nasale and footpads, of 34 cases. The literature indicates that interstitial pneumonia, type II cell hyperplasia, and inclusion bodies in airway epithelial cells, type II pneumocytes, and alveolar macrophages are commonly reported in the lungs of dogs with distemper [38,39,40,41,42,43]. Furthermore, it is noted that the immune system is suppressed during CDV infection due to the infection of approximately 80% of lymphocytes, which predisposes to secondary bacterial infections, and that *Bordetella*, adenovirus, and *Pneumocystis* may be present in the respiratory system. Secondary infections such as *Toxoplasma*, Tyzzer’s disease, and *Sarcocystis* in the nervous system, and *Cryptosporidium* and *Escherichia coli* in the gastrointestinal system have been reported [33,44].

Our findings revealed that, consistent with literature data, interstitial pneumonia and purulent bronchopneumonia were frequently observed in the lungs of many dogs. However, it was found that bronchopneumonia was generally mild or absent in areas of interstitial pneumonia, whereas in some cases the bronchopneumonia picture was quite severe and accompanied by type II pneumocyte proliferations. Secondary infections and bacterial pneumonia were detected in a portion of studied panthers, with these cases reportedly exhibiting clinical symptoms over a prolonged period [42]. Unlike the aforementioned study, bronchopneumonia was also observed in cases where clinical symptoms had recently developed. We hypothesize that an acute form of distemper, combined with possibly insufficient colostrum intake, led to secondary bacterial pneumonia as a result of marked CDV-induced immunosuppression. These observations are consistent with the lesions previously reported in the literature [42]. In this study, purulent lymphadenitis was frequently observed in cases of bronchopneumonia, along with syncytial giant cell formation in the lung in one case, and inclusion bodies within the giant cells. Furthermore, consistent with the literature [39,40,43,45,46], inclusion bodies were detected in bronchial and bronchiolar epithelial cells, type II pneumocytes, and alveolar macrophages, and it was determined that these were generally observed in areas where bronchopneumonia was absent or very mild. However, as documented in the literature, inclusions are generally absent in areas of severe bronchopneumonia; this phenomenon is attributed to inflammatory infiltration masking the inclusions within purulent lesions [42].

The affinity of CDV for lymphocytes has been widely reported in the scientific literature [39,45,47,48,49]. In this study, the thymus, spleen, mesenteric, mediastinal, and tracheobronchial lymph nodes of CDV-infected dogs were examined macroscopically and microscopically. Necropsies of CDV-positive cases revealed that the thymus was generally atrophic. In some cases, due to severe atrophy, the organ was indistinguishable from other tissues and was examined along with surrounding tissues. Microscopic evaluations showed that severe lymphoid hypoplasia was the most significant lesion in the atrophic thymus, with a disruption of the normal histological structure. In these specimens, the corticomedullary border was indistinguishable, with the thymic cortex remaining approximately 1–3 cells thick in some cases, and intranuclear inclusions in thymocytes rarely present. In studies evaluating the impact of CDV on the thymus, it has been reported that the organ shrinks five-to-six-fold by the 10th day post-infection and that the corticomedullary border disappears microscopically, which parallels the findings of the present study [50].

The current investigation demonstrated that in diseased dogs, unlike the thymus, the spleen and lymph nodes generally did not exhibit macroscopic dimensional changes but were hyperemic. In cases of secondary purulent bronchopneumonia, the mediastinal and tracheobronchial lymph nodes displayed purulent lymphadenitis, necrosis, lymphoid depletion in follicular germinal centers, and intranuclear inclusions within mononuclear leukocytes. Similar histopathological findings–namely purulent lymphadenitis, necrosis, lymphoid depletion, and intranuclear inclusions–were observed in the mesenteric lymph nodes in cases of severe purulent gastroenteritis. Some studies [33] have shown that due to CDV’s strong affinity for lymphocytes, approximately 80% of lymphocytes in infected dogs are CDV-positive, leading to significant immunosuppression. In light of this, the high incidence of purulent lymphadenitis observed in our study was interpreted as a direct consequence of CDV-induced immunosuppression.

It was noted that microscopic findings in the spleen of infected dogs generally resembled those in lymph nodes, but purulent inflammation was less pronounced. Furthermore, lymphoid depletion was observed in the follicular germinal centers of the spleen, with widespread intranuclear eosinophilic inclusion bodies, particularly in lymphocytes and macrophages. The H&E findings observed in the lymphoid tissues and organs of the affected dogs were generally consistent with the literature [39,40,45,48,49,51]. However, although some authors [45,50] reported the formation of syncytial giant cells in lymphoid tissues, syncytial giant cells were not found in lymphoid organs in this study, but were detected in the lung in only one case. This difference may be due to variations in virus strains or variability in the host immune response.

In this study, although no pathognomonic findings specific to CDV were observed in the livers of infected dogs, mild hydropic degeneration and steatosis in hepatocytes, Kupffer cell activation, sinusoidal fullness, and, in some cases, disruption of hepatic cords were detected. These findings are consistent with the literature. Beyond the detection of hepatocellular steatosis [45], studies in CDV-infected large felids have also highlighted hydropic degeneration and sinusoidal hyperemia as lesions potentially associated with the virus [49]. In our study, multifocal areas of extensive coagulation necrosis and neutrophil infiltration were also observed in the livers of two dogs that were CDV-positive by IC, RT-PCR, and IHC. The presence of widespread neutrophil infiltration in addition to necrosis was interpreted as possibly resulting from secondary bacterial infections as a consequence of severe immunosuppression in CDV infection. While the role of CDV-induced immunosuppression in secondary infections is widely recognized, research has clarified that specific parasitic findings, like trematode-related hepatic lesions, may occur independently of the viral infection [52]. This comparison shows that secondary infectious agents should also be considered in the evaluation of liver lesions.

In this study, examination of the stomach and all segments of the small intestine revealed that necrosis and desquamation were observed in the mucosal epithelia, lymphoid cell infiltrations in the lamina propria, and inclusion bodies in gastric gland epithelia, parietal cells, and infiltrating lymphocytes and macrophages. Similar microscopic lesions were generally observed in the intestinal segments, with necrosis and shedding particularly in the villous epithelia and lymphocyte infiltrations in the lamina propria; however, the presence of fewer inclusion bodies in epithelial and lymphoid cells compared to the stomach was noteworthy. Furthermore, necrosis in Peyer’s patches was observed in the ileum, and significant lymphoid depletion was detected, especially in cases with severe disease. When comparing the stomach and intestinal findings of the CDV-positive dogs in the current study with the literature, it is observed that while inclusion bodies have been reported in the stomachs of infected cases, details regarding intestinal inclusion formation were not documented [53]. In CDV-infected cases with catarrhal gastroenteritis, shedding of the intestinal and gastric mucosal epithelia has been reported, along with the presence of numerous inclusion bodies, particularly within the parietal and epithelial cells of the stomach [45]. These findings suggest that the gastric epithelium may be more susceptible to CDV infection than the intestinal epithelium. Supporting this view, inclusion bodies have been reported in the stomachs of Minsk cats [54] and in the stomach of a snow leopard [46]. These similar findings in different species indicate that the effects of CDV on the digestive system epithelium are largely conserved across species. The lower incidence of inclusions in the intestines in our study may be due to the virus replicating more limitedly in the intestinal epithelium or to the inclusions being shed before they become noticeable due to the rapid regeneration capacity of the intestinal mucosa. The lymphoid depletion in Peyer’s patches is consistent with lymphotropism of CDV and has been evaluated as a reflection of immunosuppression at the digestive system level.

In this study, while the kidneys of dogs with CDV generally showed normal histological structure, purulent and interstitial nephritis were detected in a small number of cases. In cases with severe nephritis, hyaline casts were observed in the tubular lumens, and it was determined that the main findings specific to CDV were concentrated especially in the renal pelvis. Hydropic degeneration, intranuclear and intracytoplasmic inclusion bodies, and rarely mononuclear cell infiltrations were detected in the epithelial cells of the renal pelvis. Pathological findings detected in the proximal tubule epithelia of CDV-infected large cats include severe degeneration, nonpurulent interstitial nephritis, and the formation of hyaline casts [49]. However, unlike this study, the lower incidence of tubular hyaline casts in our study was attributed to the fact that the cases were primarily in the acute stage of the disease. While mild nephritis has been detected in raccoons with CDV [52], intranuclear and intracytoplasmic inclusion bodies in renal pelvis epithelial cells have also been reported, aligning with the findings of the present study [54].

Similarly, in the bladder, hydropic degeneration in the mucosal epithelium, patchy lymphocyte infiltrations in the lamina propria, and inclusion bodies in epithelial cells were observed; it was noted that inclusions formed especially in areas where hydropic degeneration was intense. These findings are consistent with the presence of inclusion bodies in the renal pelvis and bladder epithelial cells of CDV-infected dogs in many studies [40,41,51]. Furthermore, intense hydropic degeneration has been reported in the bladder epithelium of CDV-infected pumas [55], while the presence of inclusion bodies in the bladder epithelium of CDV-infected foxes has also been documented [56]. These interspecies similarities demonstrate that the tropism of CDV for the urinary system epithelium is conserved across a wide host range. The presence of significant inclusions in the renal pelvis and bladder, even in acute cases, suggests that these areas are susceptible to viral replication and have diagnostic value.

In this study, orthokeratotic and parakeratotic hyperkeratosis was recorded as the most prominent lesion on the planum nasale and footpads of CDV-positive dogs. In addition to hyperkeratosis, hyperplasia was observed, particularly in epidermal keratinocytes, and hydropic degeneration and intranuclear and intracytoplasmic inclusion bodies were detected in keratinocytes in the hyperplastic stratum spinosum and stratum granulosum layers. In serial sections stained with H&E, the cells suspected of containing inclusion bodies were confirmed as CDV-positive by IHC, and scattered mononuclear cell infiltrations were seen in the dermal layer. These findings are largely consistent with the literature. In CDV-infected dogs, syncytial giant cell formations accompanied by hyperkeratosis, as well as both intracytoplasmic and intranuclear inclusion bodies within the cells of the Malpighi layer, have been documented [57,58]. It has also been noted that inclusions and syncytial giant cell formations occur within sweat and sebaceous glands. Although rare, intranuclear inclusions are reported to be easily distinguishable from normal cells due to characteristic chromatin condensation along the nuclear membrane [57]. However, while hyperkeratosis and dermal lymphocyte infiltrations have been reported in the footpads of CDV-positive dogs, some studies have noted an absence of inclusions in the epidermis and no microscopic changes in the sweat glands [59]. This difference may be due to variability in the virulence of the virus strain, the stage of infection, or the host immune response. The clear demonstration of inclusions in the epidermis in our study, their confirmation by IHC, and the observation of similar changes in the sweat glands suggest that the effects of CDV on the integumentary system may be more widespread than previously reported by some authors.

In this study, neuronal necrosis and neuronophagia, glial cell proliferations, demyelination, perivascular mononuclear cell infiltrations, hyperemia in the choroid plexus, and inclusion bodies in astrocytes, neurons, and ependymal cells were detected in the brain, cerebellum, brainstem, and spinal cord tissues of CDV-infected dogs. Additionally, *Toxoplasma* pseudocysts were detected in the brain of two CDV-positive cases. It has been reported that lesions in CDV-positive dogs are closely related to the duration of the disease, with acute infection characterized by white matter vacuolization, while chronic cases exhibit significant perivascular infiltrations, demyelination, and axonal loss [5]. The relatively mild CNS lesions in this study were attributed to the fact that most cases were puppies that had not received sufficient colostrum and to the acute course of the disease.

Demyelination was identified as the most significant lesion in the CNS in the present study, which is consistent with literature reports stating that it typically develops between days 21 and 24 of infection [1,36]. Experimental studies have demonstrated that demyelination manifests by day 24 post-infection, with a particular localization around the fourth ventricle [35]. Furthermore, these lesions are reported to be most prominent in regions such as the cerebellum, cerebral peduncle, and the fourth ventricle [31]. In our study, consistent with the literature [35,56,60], demyelination was found to be concentrated especially around the fourth ventricle and was also observed around the spinal cord, third ventricle, and lateral ventricle.

It has been reported that perivascular cell infiltration progressively increases with the duration of the disease, whereas such infiltrations are typically absent in acute cases [5]. In this study, since most of the cases were acute, perivascular infiltrations were observed in only a small number of cases and were of mild severity. The presence of inclusion bodies in astrocytes and ependymal cells is commonly reported in the literature [3,37,40,58,60]. In our study, consistent with these findings, inclusions were frequently observed in both astrocytes and ependymal cells, and it was noteworthy that they were more prevalent, especially in ventricular peripheries where demyelination was severe. However, inclusions were also detected in neurons in some cases; intranuclear and intracytoplasmic (often appearing as multiple granular bodies) inclusions have been observed in motor neurons in the brainstem and spinal cord. The presence of inclusion bodies in astrocytes and neurons across the cervical, thoracic, and sacral sections of the spinal cord has been documented in the literature, supporting the findings of the present study [61]. These findings demonstrate that CDV can also infect neurons and that neuronal inclusions can be observed not only in chronic or subacute cases but also in the acute phase.

In this study, numerous tissues (lungs, heart, lymph nodes, thymus, liver, stomach, pancreas, small intestines, spleen, kidney, bladder, CNS organs, footpads, and planum nasale) from 34 dogs were evaluated for CDV using the IHC method, and an immune-positive reaction was detected in 32 cases. Lungs were found to be positive in 27 cases, and the immune reaction occurred especially in type II pneumocytes, bronchial and bronchiolar mucosal epithelia, bronchial gland cells, and macrophages. CDV positivity was found in all animals with purulent or interstitial pneumonia and was predominantly observed in bronchial and bronchiolar mucosal epithelia. In addition, syncytial giant cells containing inclusion bodies were detected in one case; both these cells and the inclusions were confirmed to be CDV-positive.

CDV positivity in the lungs of different animal species with distemper has been widely reported in the literature [45,49,53,62]. In accordance with the findings of the present study, it has been reported that the CDV presence in the lungs predominantly occurs in alveolar macrophages, type II pneumocytes, bronchial and bronchiolar mucosal and glandular epithelia, and syncytial cells [44,63]. However, it has been reported that in certain wild felids infected with CDV, viral positivity within the lungs may be restricted to the bronchial and bronchiolar mucosal epithelium as well as the bronchial glands [55]. This difference may be due to variations in receptor distribution between species or differences in the tropism of the virus strain. In our study, the strong positivity detected especially in type II pneumocytes and macrophages indicates that CDV can infect a wide range of cells in lung tissue and that this plays an important role in respiratory system pathogenesis. CDV positivity in syncytial giant cells supports the idea that the virus can spread by causing cell-to-cell fusion.

In our study, CDV positivity in lymphoid tissues and organs was observed to be concentrated particularly in the germinal centers of lymph follicles, and immune-positive reactions were also detected in perifollicular lymphocytes and macrophages. The thymus was identified as the most severely affected lymphoid organ; histopathologically, differentiation between the cortex and medulla was difficult due to severe lymphocytolysis, and a severe reaction was observed especially in thymocytes in the medullary region, with almost all thymocytes being immune-positive in some cases. Histopathologically, the spleen was found to have the highest number of inclusion bodies among lymphoid organs, and similarly to other lymphoid tissues, CDV-positive reactions occurred particularly in the germinal centers of lymphoid follicles in the white pulp, with inclusion bodies in lymphocytes in the germinal centers showing strong CDV positivity in IHC staining. These findings indicate that the localization of CDV in lymphoid tissues has a distinct importance in the pathogenesis of the disease. Studies in the literature regarding the distribution of CDV in lymphoid tissues [15,40,45,49,53] are consistent with our findings, reporting CDV-positive reactions in lymphocytes and macrophages in follicular germinal centers of lymph nodes and spleen. In various reports, CDV positivity has been detected in seven out of eight spleens and six out of seven lymph nodes [53], while in other studies, it has been identified in nine out of fourteen spleens and all fourteen mesenteric lymph nodes [45]. In this study, CDV-positive reactions were detected in 20 out of 23 mediastinal and tracheobronchial lymph nodes, 16 out of 21 mesenteric lymph nodes, 21 out of 30 spleens, and 16 out of 20 thymuses. It was noted that cases with CDV-positive lungs usually also had positive mediastinal and tracheobronchial lymph nodes, while cases with positive stomach and intestinal lymph nodes mostly had positive mesenteric lymph nodes. When H&E findings in lymphoid tissues were compared with IHC results, it was determined that the inclusion rate and CDV reaction in lymphocytes were more intense in cases with lymphoid depletion. It has been reported that the CDV positivity rate increases in parallel with depletion in the spleen and lymph nodes, supporting the findings of the present study [10]. This agreement shows that the lymphocyte loss caused by CDV in lymphoid tissues is directly related to the replication intensity of the virus.

In our study, CDV immune-positive reactions were detected in Kupffer cells and bile duct epithelial cells in the liver of 18 out of 32 dogs. When CDV positivity in bile duct epithelium and epithelial cells in other organs was compared in all distemper-affected dogs, the immune reaction in bile duct epithelia was found to be weaker. The fact that all cases with CDV-positive livers also had positive stomach and intestines suggests that, in addition to general viremia, the agent may have reached the liver via the portal circulation from the digestive tract. In the literature, it has been reported that the liver was CDV-positive in 12 out of 14 dogs examined [45]. Several other authors [40,44,64] have also reported that the CDV immune-positive reaction occurs particularly in Kupffer cells and bile duct epithelia, in parallel with the findings of our study. These findings indicate that the liver is an important target organ for both the virus and the virus-dependent immune response in CDV infection. The weaker reaction in the bile duct epithelium may be due to low receptor expression levels in these cells or limited viral replication. Furthermore, the association between gastrointestinal positivity and liver positivity supports the possibility that the oral-fecal and portal routes may play a role in hepatic dissemination of CDV.

In Canine distemper virus infection, one of the most severely affected systems is the gastrointestinal system. In this study, CDV-positive reactions were detected in 23 out of 28 cases in the stomach, 18 out of 32 cases in the duodenum, 19 out of 33 cases in the jejunum, and 22 out of 32 cases in the ileum using IHC staining. Positive reactions occurred in inflammatory infiltrative cells in the mucosal lining and glandular epithelia, as well as in the lamina propria of both the stomach and intestinal segments, and also in lymphocytes in Peyer’s patches in the ileum. Inclusion bodies were rarely found in CDV-positive epithelial cells and infiltrative leukocytes, and these were also determined to be immune-positive. In the literature, CDV positivity has been reported in the gastric and intestinal mucosal epithelia of the stomach and small intestine in seven out of eight dogs [53]. Furthermore, studies have identified viral presence in 13 out of 14 stomachs and 11 out of 14 duodenums in other infected species, specifically localized within the superficial covering and glandular epithelia [45]. In this study, when evaluating CDV positivity in the small intestinal segments, mucosal surface and glandular epithelia, inflammatory infiltrations in the lamina propria, and Peyer’s patches were considered together, and it was observed that the number of cases with ileum positivity was higher compared to other segments. It was thought that this high positivity rate in the ileum may be due to the high positivity in Peyer’s patches in the region, and that Peyer’s patches may play a role in the lymphogenous spread of the virus. Indeed, the fact that in some dogs, although a CDV-positive reaction was observed in the ileal Peyer’s patches, the lamina propria and villi and crypt epithelia were found to be negative in the same intestinal segment supports this hypothesis. These findings suggest that Peyer’s patches may be an important entry point or early replication site for CDV infecting the gastrointestinal system.

In our study, CDV was observed in 20 out of 32 cases, with the exocrine pancreatic cells showing a positive immune response, while no positive reaction occurred in the endocrine pancreas (islets of Langerhans). These findings reveal that CDV has a significant affinity for the exocrine pancreas. In line with the results of the present study, immunohistochemical (IHC) positivity for CDV has been reported in the acinar cells of the exocrine pancreas in various wildlife species, including foxes, martens, and badgers [65]. Additionally, while viral presence was identified in the pancreas of nine out of fourteen examined raccoons, the specific anatomical region of the reaction was not defined in that study [45]. In contrast to the exocrine involvement, a comprehensive evaluation of 272 cases from diverse species identified a positive reaction in the Islets of Langerhans in only a single instance [49]. This single case finding suggests that endocrine pancreatic infection is a rare occurrence. The absence of positivity in the islets of Langerhans in any case in our study and the widespread positivity in the exocrine pancreas indicate that CDV primarily targets the exocrine component of the pancreas. This could be due to the virus having higher receptor expression in acinar cells than in endocrine cells, or the exocrine pancreas providing a more favorable microenvironment for viral replication.

Our study showed that the urinary system was severely affected in dogs with distemper; CDV-positive reactions were detected in the kidneys in 24 out of 34 cases and in the bladder in 19 out of 28 cases. Immune-positive reactions in the kidneys occurred especially in the epithelial cells of the renal pelvis and in the bladder in the mucosal epithelia; rarely, inflammatory infiltrative cells in both organs were found to be virus-positive. Intranuclear and intracytoplasmic inclusion bodies, frequently observed in the epithelia of the renal pelvis and bladder, were identified as the most important lesions detected in these organs. In the literature, consistent with the results of our study [15,40,53,55], CDV immunopositive staining in urinary system organs has been reported to occur predominantly in the renal pelvis and bladder epithelia. High prevalence rates of CDV have been documented in the urinary system, with reports identifying positivity in 12 out of 14 kidneys and 10 out of 12 bladder samples [45]. Similarly, other studies have established viral presence in seven out of eight kidneys and six out of seven bladder tissues [53]. The number of cases with CDV-positive reactions in the urinary system in our study (kidney: 24/34, bladder: 19/28) is similar to the rates in the literature. This consistency demonstrates that the tropism of CDV on the urinary system epithelium is highly conserved across different studies and species. The strong positivity and frequency of inclusions in the renal pelvis and bladder epithelium support the idea that these areas are reliable sampling sites for diagnosing CDV infection. Furthermore, positivity in inflammatory infiltrations suggests that the virus may also influence the local immune response in these organs.

In our study, we found that the footpads and nasal tissues of dogs of different age groups infected with CDV were significantly affected by the disease. CDV-positive reactions were observed in the footpads in 15 out of 25 cases and in the planum nasale in 15 out of 26 cases. In nasal tissue samples, the immune-positive reaction was found to be severe in the stratum spinosum, stratum granulosum, and stratum corneum layers of the epidermis, and weaker in the stratum germinativum layer compared to other layers. CDV-positive reactions in epidermal layer cells have been widely reported in the literature [53,59,62]. In the literature, CDV positivity has been identified in the epidermal layer in 9 out of 14 cases [45], as well as in seven out of eight examined cases [53]. Furthermore, consistent with the findings of the present study, it has been reported that the viral reaction is relatively weak in the basal layer of the epidermis, whereas positivity is significantly more intense within the stratum spinosum [53]. In the present study, intense CDV positivity was observed within the sweat gland epithelia of the dermis, which is consistent with previous reports of immune-positive reactions in these tissues [59]. These findings demonstrate that the integumentary system is an important target in CDV infection, both diagnostically and pathogenetically. Strong positivity in the upper layers of the epidermis suggests that the virus replicates during the keratinocyte differentiation process, while the weak reaction in the basal layer may be due to these cells being more resistant or having low receptor expression. Intense positivity in the sweat gland epithelium suggests that CDV can also infect eccrine glands and that these tissues may be valuable for diagnostic sampling.

In our study, different parts of the brain, cerebellum, brainstem, and spinal cord, which are central nervous system organs, were microscopically evaluated for CDV. In the brain, CDV immunopositive reactions were detected in astrocytes, ependymal cells lining the ventricles, choroid plexus epithelia, and in neurons in two cases. In the cerebellum, particularly in the ependymal cells lining the fourth ventricle and in the astrocytes in this region, strong positivity was observed. In the brainstem and spinal cord, unlike other parts, CDV-positive reactions were seen in motor neurons in addition to astrocytes; in some cases, it was noteworthy that motor neurons were positive while astrocytes were negative. Similarly, in the literature [40,44,53,66,67,68], CDV positivity has been reported in the CNS, usually in astrocytes, ependymal cells, choroid plexus epithelia, and rarely in neurons. In the central nervous system, CDV positivity has been identified in 11 out of 14 brain samples, 10 out of 14 cerebellum samples, and six out of eight spinal cord samples [45]. Similarly, other reports indicate viral presence across the brain, cerebellum, and brainstem in seven out of eight examined cases [53]. In this study, CDV-positive reactions were detected in 25 out of 33 cases in the brain, 27 out of 34 cases in the cerebellum, 25 out of 34 cases in the brainstem, and 17 out of 23 cases in the spinal cord.

In the present study, immune reactions were found to be most intense around the fourth ventricle, which is consistent with literature reports identifying particularly severe IHC reactions in this region [53]. Histopathologically, demyelination was generally observed around the fourth ventricle, and inclusion bodies were more prevalent in astrocytes and ependymal cells, particularly in these areas. The CDV-positive reactions observed in ependymal cells and choroid plexus epithelia around the ventricles are consistent with other studies [15,53,66,67]. The positivity in motor neurons in the brainstem and spinal cord, and in some cases the negativity of astrocytes, demonstrates that CDV can directly infect neurons and that cell tropism can vary in different CNS regions. This suggests that the virus may target different neural cell types depending on the host immune response or strain differences.

In this study, it was determined that CDV-positive astrocytes were predominantly found around the ventricles, but in some cases, they were localized around the vessels, and in some of these cases, the vascular endothelia were also CDV-positive. In the literature, it has been suggested that CDV may reach the CNS through three different mechanisms [10,36,37,69,70]: (1) the virus reaching the brain via the bloodstream, passing through the endothelium and infecting the astrocytes; (2) spreading to the CNS via the CSF by infecting the choroid plexus epithelia; (3) the virus reaching the brain via the olfactory bulb after being inhaled.

Different mechanisms have been proposed for the neuroinvasion of CDV. It has been suggested that viruses ingested nasally can reach the brain via the olfactory bulb to infect neurons [70]. Alternatively, the virus may infect the CNS through interaction with endothelial cells via the adhesion of infected cells and leukocytes across the blood-brain barrier [69]. Furthermore, another pathway involves the infection of the choroid plexus epithelium, followed by passage into the CSF, reaching ependymal cells, and subsequently infiltrating the CNS parenchyma [10,36,37]. In the present study, the diffuse CDV positivity observed in vascular endothelia and surrounding astrocytes within CNS organs supports the hypothesis of infection via the vascular endothelium [69]. Furthermore, the identification of CDV positivity restricted to the choroid plexus epithelia and ependymal cells in certain offspring, notably those lacking neurological symptoms and having had insufficient colostrum intake, aligns with the proposed mechanism of spread via the choroid plexus-CSF axis [10,36,37]. However, as the olfactory bulb was not examined in this study, no conclusions could be drawn regarding the olfactory pathway suggested in the literature [70].

It has been proposed that astrocytes serve as reservoirs during CDV infection, facilitating the infection of neurons via infected glial cells [71]. A similar mechanism has been described in human measles virus infection, where astrocytes act as reservoirs for neuronal involvement [72]. Additionally, high viral positivity in the nasal cavity is suggested to allow CNS entry through synaptic connections involving olfactory receptor neurons, glomeruli, and mitral cells [10,70], a hypothesis experimentally validated in ferrets. Furthermore, it has been emphasized that the measles virus can spread between neurons via synapses, suggesting the potential existence of currently unidentified receptors in synaptic regions [73]. In six cases within our study, astrocytes in the brainstem and the cervical, thoracic, and lumbar spinal cord segments were observed to be CDV-negative, whereas corresponding motor neurons demonstrated distinct immunopositivity. Based on these descriptive findings, it could be cautiously hypothesized that CDV may disseminate within the CNS independently of astrocytes, potentially utilizing neuroanatomical pathways. This pattern raises the possibility of trans-synaptic transmission between neurons and may point toward the involvement of alternative receptors or pathways.

Numerous studies have been conducted to date on the pathogenesis of canine distemper, with particular emphasis on the CD150 and Nectin-4 receptors, which the virus uses to enter host cells. However, it is noteworthy that most research on these receptors has been conducted at the cell culture level, and studies using naturally occurring distemper-infected dogs are quite limited. While there are a few studies in the scientific literature regarding the Nectin-4 receptor in CDV-infected dogs [10,23,24,74], there is no data on the identification of cells carrying the CD150 receptor; information on this subject is generally based on studies conducted on cells infected with the measles virus in humans. The CD150 receptor has been reported to be present in immature thymocytes, activated T and B lymphocytes, blood monocytes, tissue macrophages, and dendritic cells [19,20,21,22,75].

In this study, CD150 receptor expression was investigated using the IHC method in naturally infected dogs with CDV. Consistent with literature data, lymphocytes in the thymus, spleen, respiratory and digestive system lymph nodes, and Peyer’s patches, as well as macrophages in various tissues and organs, were found to be CD150 immune-positive. Furthermore, CD150 immune-positive reactions were observed in infiltrative lymphocytes and macrophages in the lamina propria of the stomach and intestines, lymphocytes in Peyer’s patches in the ileum, a small number of inflammatory cells in the footpads and dermis of the planum nasale, inflammatory infiltrative cells in the renal pelvis and lamina propria of the bladder, and lymphoid cells in the CNS. When evaluating the cell types expressing the CD150 receptor in the organs and tissues examined in this study, a high degree of agreement was found with CD150-positive cells identified in human research on this receptor. These findings indicate that CD150 expression is conserved across species in naturally infected dogs and that lymphoid and macrophage cells, in particular, play a significant role in CDV pathogenesis.

In our study, IHC staining for Nectin-4 revealed that many epithelial cells were immune-positive. Bronchial and bronchiolar mucosal epithelial cells in the lungs, mucosal lining epithelium and parietal cells in the stomach, villus and crypt epithelia in the small intestine, bile duct epithelia in the liver, epidermal squamous cells in the footpads and planum nasale, and sweat gland epithelia were found to be Nectin-4 positive. Renal pelvis and some tubule epithelia in the kidneys, and pseudostratified epithelial cells of the bladder were also found to be Nectin-4 positive. Furthermore, Nectin-4 immune-positive reactions were observed in choroid plexus epithelial cells, ependymal cells, motor neurons, and vascular endothelia in the central nervous system (CNS). The findings of the present study demonstrate a high degree of consistency with the literature. It has been documented that the Nectin-4 receptor is localized within the bronchial and bronchiolar mucosal epithelia, lamina epithelialis of the stomach and intestines, mucosal covering cells of the renal pelvis and bladder, tonsillar epithelia, epidermal keratinocytes, and specific CNS cells such as neurons and ependymal cells [10,23,24,25]. Significantly, the identification of Nectin-4 positivity within the bile duct epithelium, sweat gland epithelia, and vascular endothelia in this study suggests that the tissue distribution of this receptor may be more extensive than previously reported. The widespread expression of Nectin-4 in epithelial cells plays an important role in explaining CDV’s epithelial tropism and multisystemic infection capacity. The positivity, particularly in motor neurons and ependymal cells in the CNS, suggests that the virus can directly enter these cells and that Nectin-4 may be a potential receptor for neurological involvement.

A review of the literature reveals that the number of studies evaluating CD150 and Nectin-4 receptors and CDV using double or triple staining techniques via immunofluorescence is quite limited. Therefore, in our study, triple indirect immunofluorescence staining was applied for the first time using commercial antibodies against CD150 and Nectin-4 receptors and CDV. The presence of CDV in cells carrying these receptors was revealed, and an attempt was made to shed light on whether the virus uses these receptors to enter target cells.

In triple IF staining, it was determined that infiltrative lymphocytes and macrophages in the lungs were positive for CD150 and CDV, while bronchial and bronchiolar mucosal epithelia, bronchial gland cells, and type II pneumocytes were positive for Nectin-4 and CDV. Immunohistochemical analysis has demonstrated that CD150 expression is particularly intense around the bronchioles in CDV-infected dogs. The immune-positive cells identified in these regions are morphologically consistent with lymphocytes and macrophages [76]. In alignment with the findings of the present study, research employing IHC and dual IF techniques has demonstrated that the respiratory mucosal epithelia are positive for both CDV and Nectin-4 [23,74]. These findings show the distinct tissue localization of CDV alongside CD150 in lymphoid cells and Nectin-4 in epithelial cells, which is consistent with the established receptor pathways driving viral pathogenesis in the respiratory system. Furthermore, the successful application of the triple IF method in naturally infected dog tissues demonstrates that these techniques are reliable and informative in CDV receptor studies.

In our study, triple IF staining revealed that mucosal surface and glandular epithelia in the digestive tract were positive for Nectin-4 and CDV; infiltrative lymphocytes and macrophages in the lamina propria and lymphocytes in Peyer’s patches were positive for CD150 and CDV. In the urinary system, the mucosal epithelia of the renal pelvis and bladder were particularly positive for Nectin-4 and CDV, but some tubular epithelia were Nectin-4 positive but CDV negative.

The findings of the present study are consistent with the literature, which has documented immunopositive reactions in the epithelial lining cells of both digestive and urinary system organs using double IF staining for CDV and Nectin-4 [23,74]. Our study confirms these findings with the triple IF method and also demonstrates for the first time the coexistence of CD150 and CDV in lymphocytes and macrophages in the digestive tract. The negativity of CDV despite Nectin-4 positivity in renal tubular epithelia suggests that although these cells carry the receptor, viral infection did not occur due to a deficiency of other cellular factors, or that the viral load in these regions was too low to be detected by IHC/IF. In conclusion, Nectin-4-mediated CDV infection in digestive and urinary system epithelia and CD150-mediated CDV infection in lymphoid and macrophage cells explain the tropism and multisystemic spread of the virus in these systems.

Studies in the literature using cell cultures in humans [75], distemper-infected dogs [18], and mice [77] have reported that macrophages and lymphocytes carry the CD150 receptor. In our study, triple IF staining of lymph nodes and the spleen revealed that lymphocytes in follicular germinal centers and perifollicular macrophages, and thymocytes in the thymus, were positive for CD150 and CDV. These results are consistent with documented evidence that CDV exhibits a strong tropism for lymphocytes, where the CD150 receptor is widely recognized as a primary gateway for cellular entry [17].

Based on immunohistochemical (IHC) findings, it has been reported that CD150 immune-positive reactions occur within the lymph nodes, with a particular concentration in the parafollicular areas [76]. In contrast to these results, our study determined that CD150- and CDV-positive reactions predominantly occurred in lymphocytes in follicular germinal centers; while macrophages in perifollicular and parafollicular areas were rarely positive for both CDV and CD150. This difference may stem from differences in the sensitivity of the techniques used (IF vs. IHC), the specificity of antibody recognition, or variations in the infection stage (acute/chronic) of the cases. The strong positivity in germinal centers in our study indicates that CD150 expression, and therefore CDV infection, is more pronounced in these regions where active B cell proliferation is intense. Furthermore, the CD150/CDV positivity of thymocytes in the thymus medulla confirms that immature thymocytes are also targets for the virus. These findings confirm the critical role of the CD150 receptor in the pathogenesis of CDV in lymphoid tissues using the triple IF method in naturally infected canine tissues.

In our study, epidermal keratinocytes and sweat gland epithelia in the nasal and footpads were found to be positive for both Nectin-4 and CDV. In an immunohistochemical study, it has been reported that while epidermal cells in the footpads are CDV-positive, they remain CD150-negative [76]. This finding suggests that the virus may utilize a receptor other than CD150 for entry into the epidermal keratinocytes, where it exhibits tropism. Using both IHC and double IF methods, it has been documented that the epidermis and glandular epithelia in CDV-infected cases are positive for both CDV and Nectin-4 [78]. In alignment with these reports, the double positivity identified in the epidermal keratinocytes of the footpads and nasal tissues of infected dogs supports the involvement of the Nectin-4 receptor in the viral tropism of these specific target cells.

In the central nervous system, choroid plexus epithelia, ependymal cells, and some motor neurons were found to be positive for Nectin-4 and CDV, but astrocytes, despite being strongly positive for CDV, were negative for both Nectin-4 and CD150 receptors. In dogs infected with distemper, immunohistochemical (IHC) analysis has demonstrated CD150 immunopositivity within the brainstem and cerebellar folia. This reactivity is particularly concentrated in the substantia alba surrounding the fourth ventricle, specifically in areas characterized by lesions and CDV positivity [76]. In our study, however, despite widespread CDV positivity in astrocytes, these cells were found to be negative for CD150 and Nectin-4 receptors. These findings suggest that CDV infection in astrocytes occurs independently of these two known receptors, via an as yet unidentified alternative receptor or a different entry mechanism.

In line with our study results, the literature [23,74] also reports that choroid plexus epithelia and ependymal cells in the CNS, as well as motor neurons in the brainstem and spinal cord, are Nectin-4 and CDV-positive, while astrocytes are CDV-positive but Nectin-4-negative. Consistent with the findings of the present study, Nectin-4 positivity has been documented in the ependymal cells of the brain and cerebellum, particularly those lining the ventricular spaces [12]. These data indicate that CDV cell tropism within the CNS differs according to receptor distribution. While ependymal cells and choroid plexus epithelium are susceptible to Nectin-4-mediated infection, the intense CDV replication in astrocytes necessitates the existence of alternative entry pathways. This situation points to a significant gap in understanding the pathogenesis of CDV in the CNS and underlines the need to investigate potential novel pathways involved in astrocyte infection.

In our study, it was determined that vascular endothelia in all organs examined were positive for Nectin-4 and CDV, while blood monocytes located in the vascular lumen were positive for CD150 and CDV. In the literature, CD150-positive cells have been rarely identified within the vascular walls of healthy canine brains [74]. Conversely, vascular endothelia in the brain have been reported to express Nectin-4 [12]. Furthermore, evidence shows that dermal capillary endothelia in the skin can be positive for both Nectin-4 and CDV [78]. Based on these findings, it was interpreted that in our study, monocytes in the vascular lumen, after infection via the CD150 receptor, transport the virus to tissues and organs via the bloodstream; and vascular endothelia, by allowing entry to CDV via the Nectin-4 receptor, play an important role in the spread of the virus to all tissues and organs, especially in overcoming the blood-brain barrier in CNS infections.

In our study, using double IF staining with CDV and GFAP antibodies, it was revealed that the infected cells that were immune-positive for both markers in CNS organs were astrocytes. Literature [10,11,12,13] reports that CDV reaction in the CNS occurs especially in astrocytes, but these cells do not carry CD150 and Nectin-4 receptors. Indeed, in our study, triple IF staining with CDV, CD150, and Nectin-4 antibodies showed that astrocytes were CDV-positive but CD150- and Nectin-4-negative, consistent with the literature. Double IHC staining for CDV and GFAP has revealed that 95% of CDV-infected cells are GFAP-positive astrocytes, although this proportion decreases to 65% within demyelinated areas. Based on morphological evaluation, CDV-positive but GFAP-negative cells have been identified as likely representing macrophages and oligodendroglial cells [68]. Furthermore, studies using IF and IHC techniques [68,74,79] have reported that the primary target cells of CDV in the CNS are GFAP-positive astrocytes, as also observed in our study. These findings strongly suggest that CDV preferentially infects astrocytes in the CNS and that this infection occurs independently of the known CD150 and Nectin-4 receptors.

## 5. Conclusions

In this study, clinical, macroscopic, and microscopic findings and the organ distribution of lesions in naturally infected dogs with CDV were revealed, and it was determined that the virus infects numerous tissues and organs in different systems. The presence of CDV in tissues and organs was investigated using IC, RT-PCR, and IHC methods; RT-PCR and IHC were found to be equally sensitive, and both techniques were more sensitive than the IC method.

IHC staining revealed that CDV infects many systems, particularly epithelial cells, lymphocytes, and macrophages, and in the CNS, astrocytes, ependymal cells, choroidal plexus epithelia, and motor neurons. Triple immunofluorescence staining for CDV, CD150, and Nectin-4 showed that lymphocytes and macrophages were positive for CD150 along with CDV, while epithelial cells were positive for Nectin-4 along with CDV. These findings indicate that infected cells express the corresponding receptors, demonstrating a clear co-localization of CDV with Nectin-4 in epithelial cells and CD150 in lymphocytes and macrophages, which aligns with the established receptor-dependent tropism of the virus.

In contrast, although the CDV immune-positive reaction in the CNS is most intense in astrocytes, these cells were found to be negative for CD150 and Nectin-4 receptors. Double immunofluorescence staining with GFAP and CDV antibodies definitively confirmed that the CDV-positive but CD150- and Nectin-4-negative cells in the CNS were astrocytes. Therefore, it is thought that the virus may have entered astrocytes, the most frequently infected cells in the CNS, via alternative receptors or pathways other than CD150 and Nectin-4, as also indicated in the literature.

Furthermore, in six cases, CDV immunopositivity was observed only in motor neurons across different regions of the CNS, without a positive reaction in astrocytes. This distinctive morphological pattern leads to the cautious hypothesis that the virus may spread in the CNS independently of astrocytes, possibly via interneuronal synaptic connections.

This study is the first to apply triple immunofluorescence staining using antibodies against the virus and its two known receptors (CD150 and Nectin-4) in naturally occurring CDV-infected dogs, and provides significant contributions to future research into the pathogenesis of morbilliviruses.

## Figures and Tables

**Figure 1:**
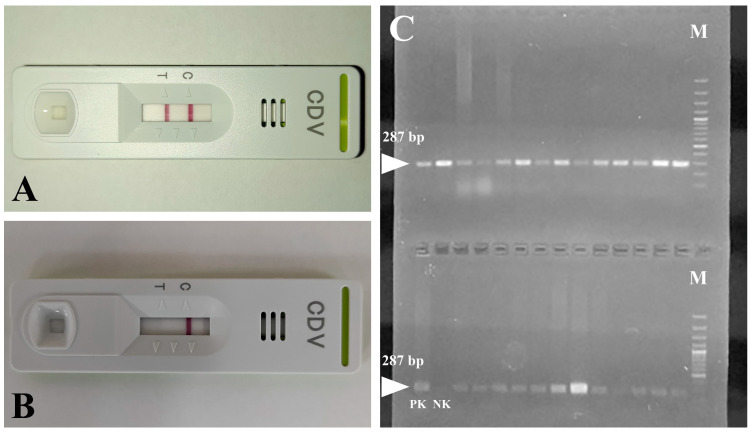
IC and PCR results. (**A**) Positive CDV test result using the IC method. (**B**) Negative CDV test result using the IC method. (**C**) Gel images obtained by running the amplicons on the samples. PK: Positive Control, NK: Negative Control, M: Ladder (100 bp). Arrowheads indicate 287 bp CDV positive gel bands.

**Figure 2:**
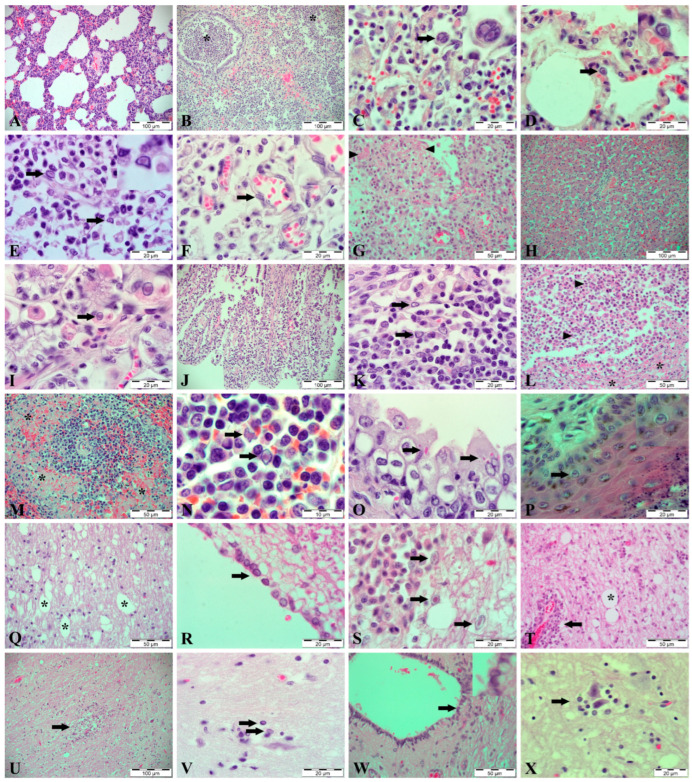
H&E images of CDV-infected dogs. (**A**) Interstitial pneumonia in the lung and infiltration in the alveolar septum, H&E, Bar: 100 µm. (**B**) Neutrophilic leukocyte infiltrations in the bronchiolar and alveolar lumens (stars) in the lung with purulent bronchopneumonia, H&E, Bar: 100 µm. (**C**) Syncytial giant cell (arrow) and intranuclear inclusion bodies (inset) in the lung, H&E, Bar: 20 µm. (**D**) Intranuclear inclusion body and chromatin margination (arrow) in a type II pneumocyte in the lung (inset), H&E, Bar: 20 µm. (**E**) Intranuclear inclusion bodies (arrows) in lymphocytes in the tracheobronchial lymph node (inset), H&E, Bar: 20 µm. (**F**) Intranuclear inclusion body (arrow) in the vascular endothelium in the tracheobronchial lymph node (inset), H&E, Bar: 20 µm. (**G**) Diffuse necrotic areas (arrowheads) in the thymus of a CDV-infected dog, H&E, Bar: 50 µm. (**H**) Mild sinusoidal hyperemia and Kupffer cell activation in the liver, H&E, Bar: 100 µm. (**I**) Intranuclear inclusion body (arrow) in the gastric glandular epithelia (inset), H&E, Bar: 20 µm. (**J**) Desquamation in the villous epithelia and inflammatory lymphocyte infiltration in the (**L**). propria in the jejunum, H&E, Bar: 100 µm. (**K**) Intranuclear inclusion bodies (arrows) in lymphocytes in ileal Peyer’s patches, H&E, Bar: 20 µm. (**L**) Necrosis (stars) and neutrophilic exudation (arrowheads) in the mesenteric lymph node, H&E, Bar: 50 µm. (**M**) Necrotic areas (stars) in the lymph follicle in the spleen, H&E, Bar: 50 µm. (**N**) Intranuclear inclusion bodies (arrows) in lymphocytes in the spleen, H&E, Bar: 20 µm. (**O**) Intracytoplasmic eosinophilic inclusion bodies (arrows) in the epithelial cells of the urinary bladder, H&E, Bar: 20 µm. (**P**) Intranuclear inclusion bodies (arrow) in epidermal keratinocytes in the footpad (inset), H&E, Bar: 20 µm. (**Q**) Demyelination (stars) in the substantia alba around the fourth ventricle in the cerebellum, H&E, Bar: 50 µm. (**R**) Intranuclear inclusion bodies (arrows) in ependymal cells surrounding the fourth ventricle, H&E, Bar: 20 µm. (**S**) Intranuclear inclusion bodies (arrows) and chromatin margination in astrocytes in the cerebellum, H&E, Bar: 20 µm. (**T**) Perivascular mononuclear cell infiltration (arrow) and demyelination (star) in the cerebellum, H&E, Bar: 50 µm. (**U**): Focal necrosis (arrow) in the brainstem, H&E, Bar: 200 µm. (**V**) Neuronophagia (arrow) in the lumbar spinal cord. H&E, Bar = 20 µm. (**W**) Intranuclear inclusion body (arrow) in the epithelial cell of the canalis centralis in the lumbar spinal cord (inset), H&E, Bar: 50 µm. (**X**) Neuronophagia (arrow) in the lumbar spinal cord, H&E, Bar: 20 µm.

**Figure 3:**
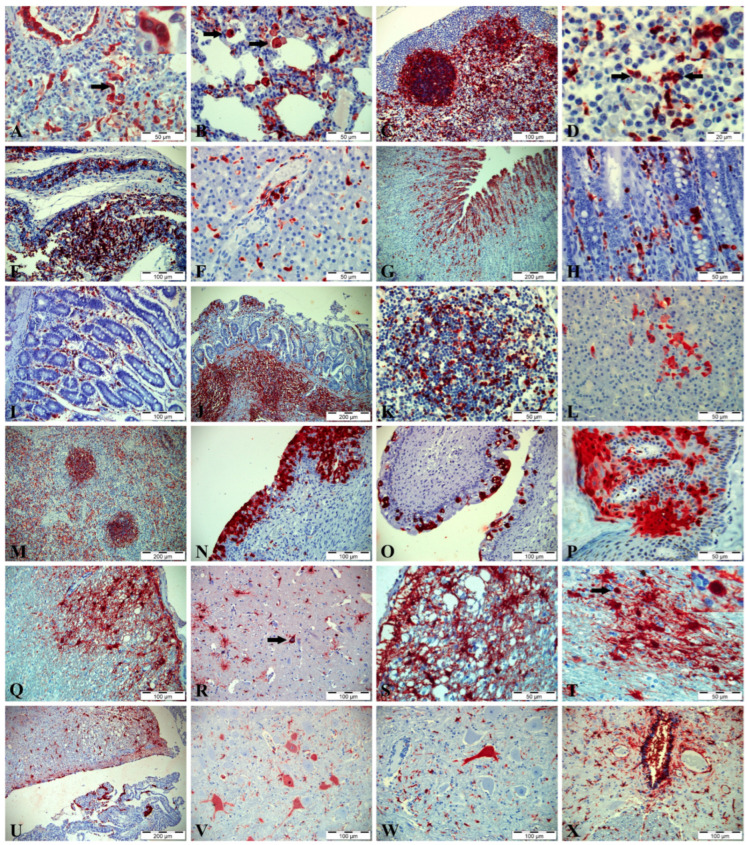
IHC images for CDV in organs and tissues of CDV-infected dogs. (**A**) CDV-positive reaction in type II pneumocytes and bronchiolar mucosal epithelia in the lung, and intranuclear inclusion body in a type II pneumocyte (arrow) (inset), (IHC) Bar: 50 µm. (**B**) CDV-positive reaction in alveolar macrophages (arrows), (IHC) Bar: 50 µm. (**C**) CDV-positive reaction in the follicle and perifollicular lymphocytes and macrophages in the tracheobronchial lymph node, (IHC) Bar: 100 µm. (**D**) CDV-positive intranuclear inclusion bodies (arrows) in lymphocytes in the tracheobronchial lymph node (inset), (IHC) Bar: 100 µm. (**E**) CDV-positive reaction in thymocytes in the thymus, (IHC) Bar: 100 µm. (**F**) CDV-positive reaction in Kupffer cells, vascular endothelia, and bile duct epithelia in the liver (IHC). Bar: 50 µm. (**G**) CDV-positive reaction in the *L. epithelialis* in the stomach, (IHC) Bar: 200 µm. (**H**) CDV-positive reaction in the villous epithelia in the duodenum, (IHC) Bar: 50 µm. (**I**) CDV-positive reaction in lymphocytes in the *L. propria* in the jejunum, (IHC) Bar: 100 µm. (**J**) CDV-positive reaction in lymphoid cells in Peyer’s patches and villous epithelia in the ileum (IHC). Bar: 200 µm. (**K**) CDV-positive reaction in lymphocytes in the follicular germinal center in the mesenteric lymph node, (IHC) Bar: 50 µm. (**L**) CDV-positive reaction in exocrine pancreatic cells, (IHC) Bar: 50 µm. (**M**) Severe CDV-positive reaction in the germinal centers of lymphoid follicles and perifollicular lymphocytes and macrophages in the spleen, (IHC) Bar: 200 µm. (**N**) Severe CDV-positive reaction in renal pelvis epithelial cells, (IHC) Bar: 50 µm. (**O**) CDV-positive reaction in the urinary bladder mucosal epithelia, (IHC) Bar: 100 µm. (**P**) CDV-positive reaction in the epidermal layer in the nose, (IHC) Bar: 50 µm. (**Q**) CDV-positive reaction in the demyelinated area around the ventriculus lateralis in the brain, (IHC) Bar: 100 µm. (**R**) CDV-positive reaction in astrocytes and a neuron (arrow) in the brain, (IHC) Bar: 100 µm. (**S**) CDV-positive reaction around the 4th ventricle in the cerebellum, (IHC) Bar: 50 µm. (**T**) CDV-positive reaction in astrocytes with an intranuclear inclusion body (arrow) in the cerebellum (inset), (IHC) Bar: 50 µm. (**U**) CDV-positive reaction in choroid plexus epithelial cells, ependymal cells, and astrocytes with demyelination around the 4th ventricle near the cerebellum (IHC). Bar: 200 µm. (**V**) Mild CDV-positive reaction in motor neurons and astrocytes in the brainstem, (IHC) Bar: 100 µm. (**W**) CDV-positive reaction in a motor neuron and astrocytic processes in the lumbar spinal cord, (IHC) Bar: 50 µm. (**X**) Severe CDV-positive reaction in canalis centralis epithelia, astrocytes, and vascular endothelia in the lumbar spinal cord (IHC). Bar: 100 µm.

**Figure 4:**
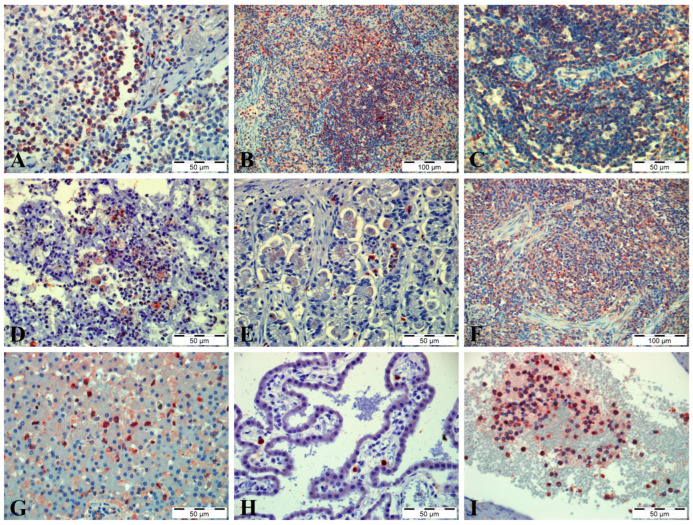
IHC images for CD150 in organs and tissues of CDV-infected dogs. (**A**) Cytoplasmic CD150-positive reaction in lymphocytes in the tracheobronchial lymph node, (IHC) Bar: 50 µm. (**B**) Diffuse CD150-positive reaction in lymphocytes in the white pulp in the spleen, (IHC) Bar: 100 µm. (**C**) CD150-positive reaction in lymphocytes around the a. centralis in the follicle in the spleen, (IHC) Bar: 50 µm. (**D**) CD150-positive reaction in alveolar macrophages in the lung, (IHC) Bar: 50 µm. (**E**) CD150-positive reaction in inflammatory infiltrative cells in the *L. propria* in the stomach, (IHC) Bar: 50 µm. (**F**) Diffuse CD150-positive reaction in lymphocytes in Peyer’s patches in the ileum (IHC). Bar: 100 µm. (**G**) CD150-positive reaction in Kupffer cells in the liver, (IHC) Bar: 50 µm. (**H**) CD150-positive reaction in a small number of inflammatory infiltrative lymphocytes in the choroid plexus in the fourth ventricle, (IHC) Bar: 50 µm. (**I**) CD150-positive reaction in mononuclear leukocytes in the vascular lumen, (IHC) Bar: 50 µm.

**Figure 5:**
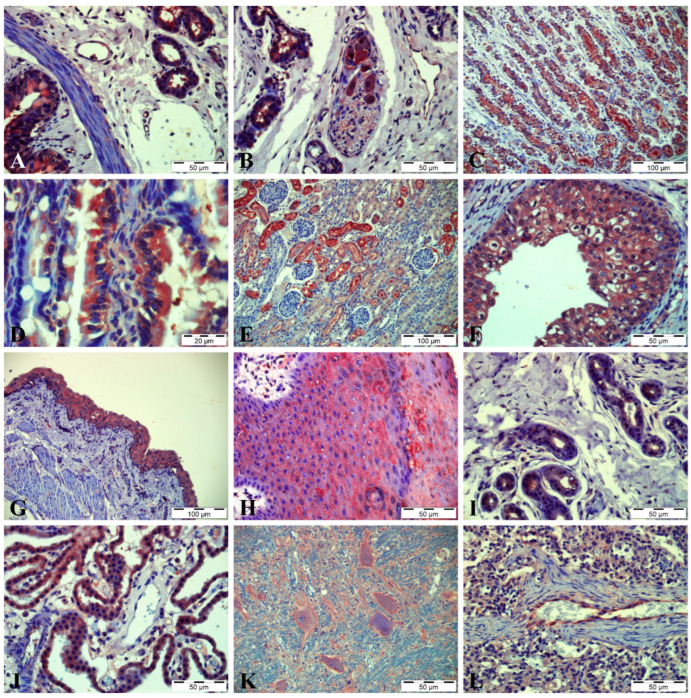
IHC images for Nectin-4 in organs and tissues of CDV-infected dogs. (**A**) Nectin-4 positive reaction in bronchial mucosal epithelia and bronchial glands in the lung, (IHC). Bar: 50 µm. (**B**) Nectin-4 positive reaction in neurons located at peripheral nerve endings and vascular endothelia in the lung, (IHC) Bar: 50 µm. (**C**) Nectin-4 positive reaction in glandular epithelia in the stomach, (IHC) Bar: 100 µm. (**D**) Nectin-4 positive reaction in villous epithelial cells in the jejunum, (IHC) Bar: 50 µm. (**E**) Nectin-4 positive reaction in proximal tubule epithelia in the renal cortex, (IHC) Bar: 100 µm. (**F**) Nectin-4 positive reaction in renal pelvis epithelia, (IHC) Bar: 50 µm. (**G**) Nectin-4 positive reaction in the *L. epithelialis* in the urinary bladder, (IHC) Bar: 100 µm. (**H**) Nectin-4 positive reaction in all epidermal layer cells in the planum nasale, (IHC) Bar: 50 µm. (**I**) Nectin-4 positive reaction in glandular epithelial cells of the footpad, (IHC) Bar: 50 µm. (**J**) Nectin-4 positive reaction in choroid plexus epithelia located in the fourth ventricle, (IHC) Bar: 50 µm. (**K**) Nectin-4 positive reaction on the membranes of motor neurons in the brainstem, (IHC) Bar: 50 µm. (**L**) Nectin-4 positive reaction in vascular endothelia in the spleen, (IHC) Bar: 50 µm.

**Figure 6:**
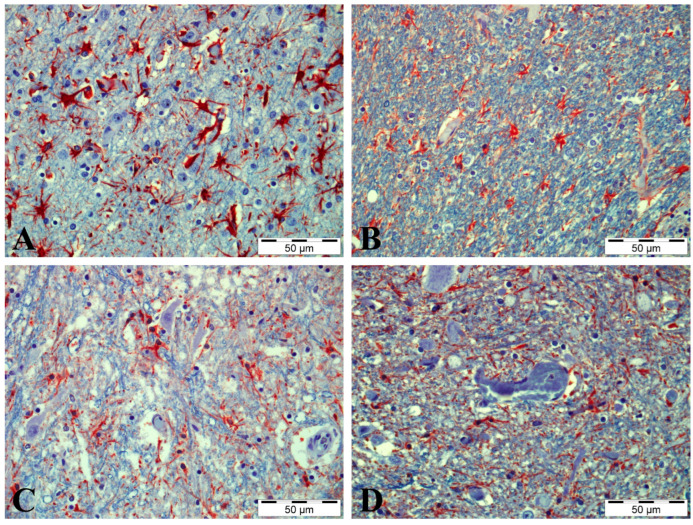
IHC images for GFAP in the brain, cerebellum, and spinal cord. (**A**) GFAP-positive reaction in astrocytes in the brain, (IHC) Bar: 50 µm. (**B**) GFAP-positive reaction in astrocytes in the cerebellum, (IHC) Bar: 50 µm. (**C**) GFAP-positive reaction in astrocytes in the cervical spinal cord, (IHC) Bar: 50 µm. (**D**) GFAP-positive reaction in astrocytes in the thoracic spinal cord, (IHC) Bar: 50 µm.

**Figure 7:**
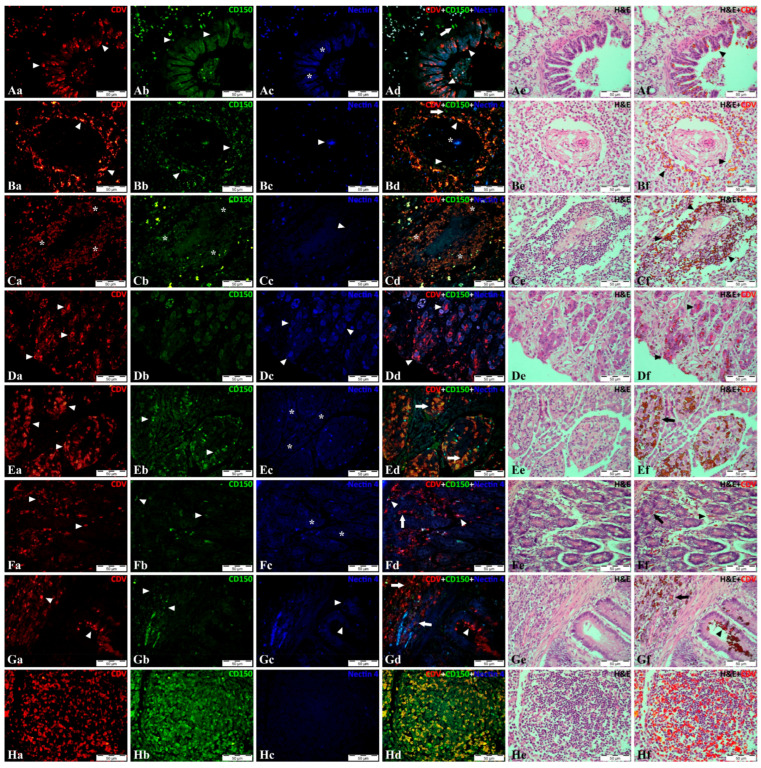
Triple IF images for CDV, CD150, and Nectin-4 in organs and tissues of the dogs within the scope of the study. (**A**) Triple IF staining images for CDV, CD150, and Nectin-4 in the lung. (**Aa**) CDV-positive reaction in bronchial mucosal epithelia (arrowheads), (IF) Bar: 50 µm. (**Ab**) CD150-positive reaction in some peribronchiolar cells (arrowheads), (IF) Bar: 50 µm. (**Ac**) Nectin-4 positive reaction in bronchial mucosal epithelia (stars), (IF) Bar: 50 µm. (**Ad**) Merged image of CDV, CD150, and Nectin-4; CDV and Nectin-4 positive reaction in bronchial mucosal epithelia (arrowheads), and CD150-positive but CDV and Nectin-4-negative cells around the bronchus (arrow), (IF) Bar: 50 µm. (**Ae**) H&E-stained image of the same section subjected to IF, (H&E) Bar: 50 µm. (**Af**) CDV-positive areas in bronchiolar epithelia in the section stained with H&E (arrowhead), (H&E + IF) Bar: 50 µm. (**B**) Triple IF staining images for CDV, CD150, and Nectin-4 in the tracheobronchial lymph node. (**Ba**) CDV-positive reaction in the perivascular area in the lymph node (arrowheads), (IF) Bar: 50 µm. (**Bb**) CD150-positive reaction in perivascular cells in the lymph node (arrowheads), (IF) Bar: 50 µm. (**Bc**) Nectin-4 positive areas in the vascular endothelium in the lymph node (arrowhead), (IF) Bar: 50 µm. (**Bd**) Merged image of CDV, CD150, and Nectin-4 IF in the tracheobronchial lymph node; CDV and CD150 positive lymphocytes (arrowheads), CD150-positive but CDV-negative lymphocyte (arrow), and Nectin-4 positive but CDV-negative vascular endothelia (star), (IF) Bar: 50 µm. (**Be**) H&E staining of the same section subjected to IF in the lymph node, (H&E) Bar: 50 µm. (**Bf**) CDV-positive reaction in lymphoid cells in the section stained with H&E in the lymph node (arrowheads), (H&E + IF) Bar: 50 µm. (**C**) Triple IF staining images for CDV, CD150, and Nectin-4 in the thymus. (**Ca**) CDV-positive reaction in perivascular thymocytes (star), (IF) Bar: 50 µm. (**Cb**) CD150-positive areas in perivascular thymocytes (star), (IF) Bar: 50 µm. (**Cc**) Nectin-4 positive reaction in the vascular intima in the same region (arrowhead), (IF) Bar: 50 µm. (**Cd**) Merged image of CDV, CD150, and Nectin-4 in the thymus, resulting in both CDV- and CD150-positive thymocytes (star), (IF) Bar: 50 µm. (**Ce**) H&E-stained image of the same section subjected to IF, (H&E) Bar: 50 µm. (**Cf**) CDV-positive thymocytes in the section stained with H&E in the thymus (arrowheads), (H&E + IF) Bar: 50 µm. (**D**) Triple IF staining images for CDV, CD150, and Nectin-4 in the stomach. (**Da**) CDV-positive reaction in gastric glandular epithelia (arrowheads), (IF) Bar: 50 µm. (**Db**) CD150-negative reaction in the stomach, (IF) Bar: 50 µm. (**Dc**) Nectin-4 positive cells in glandular epithelia (arrowheads), (IF) Bar: 50 µm. (**Dd**) Merged image of CDV, CD150, and Nectin-4; Nectin-4 and CDV-positive areas (arrowheads), (IF) Bar: 50 µm. (**De**) H&E-stained image of the same section subjected to IF, (H&E) Bar: 50 µm. (**Df**) CDV-positive glandular epithelia in the section stained with H&E in the stomach (arrowhead), (H&E + IF) Bar: 50 µm. (**E**) Triple IF staining images for CDV, CD150, and Nectin-4 in the duodenum. (**Ea**) CDV immunopositive areas in the *L. propria* (arrowheads), (IF) Bar: 50 µm. (**Eb**) CD150-positive reaction in inflammatory infiltrative cells in the *L. propria* (arrowheads), (IF) Bar: 50 µm. (**Ec**) Nectin-4 positive areas in villous epithelia (stars), (IF) Bar: 50 µm. (**Ed**) Merged image of CDV, CD150, and Nectin-4: CDV and CD150 positive areas (arrows), (IF) Bar: 50 µm. (**Ee**) H&E-stained image of the same section subjected to IF, (H&E) Bar: 50 µm. (**Ef**) CDV-positive reaction in inflammatory infiltrative cells in the section stained with H&E (arrow), (H&E + IF) Bar: 50 µm. (**F**) Triple IF staining images for CDV, CD150, and Nectin-4 in the jejunum. (**Fa**) CDV-positive reaction in epithelial cells and infiltrative inflammatory cells (arrowheads), (IF) Bar: 50 µm. (**Fb**) CD150-positive reaction in a small number of inflammatory infiltrative cells (arrowheads), (IF) Bar: 50 µm. (**Fc**) Nectin-4 positive staining in epithelial cells in the jejunum (stars), (IF) Bar: 50 µm. (**Fd**) Merged image of CDV, CD150, and Nectin-4 in the jejunum; CD150- and CDV-positive inflammatory infiltrative cells (arrowheads), and Nectin-4 and CDV-positive epithelial cells (arrow), (IF) Bar: 50 µm. (**Fe**) H&E-stained image of the same section subjected to IF, (H&E) Bar: 50 µm. (**Ff**) CDV-positive areas in the section stained with H&E in the jejunum; crypt epithelia (arrow) and lymphocytes (arrowhead), (H&E + IF) Bar: 50 µm. (**G**) Triple IF staining images for CDV, CD150, and Nectin-4 in the ileum. (**Ga**) CDV-positive reaction in lymphocytes and crypt epithelia in Peyer’s patches (arrowheads), (IF) Bar: 50 µm. (**Gb**) CD150-positive reaction in lymphocytes in Peyer’s patches (arrowheads), (IF) Bar: 50 µm. (**Gc**) Nectin-4 positive reaction in crypt epithelia in the ileum (arrowheads), (IF) Bar: 50 µm. (**Gd**) Merged image of CDV, CD150, and Nectin-4 in the ileum, resulting in CDV and CD150 positive lymphocytes (arrows) and Nectin-4 and CDV-positive crypt epithelia (arrowhead), (IF) Bar: 50 µm. (**Ge**) H&E-stained image of the same section subjected to IF, (H&E) Bar: 50 µm. (**Gf**) CDV-positive reaction in lymphocytes (arrow) and crypt epithelia (arrowhead) located in Peyer’s patches in the section stained with H&E, (H&E + IF) Bar: 50 µm. (**H**) Triple IF staining images for CDV, CD150, and Nectin-4 in the mesenteric lymph node. (**Ha**) CDV-positive lymphocytes and macrophages in the lymph node, (IF) Bar: 50 µm. (**Hb**) Positive staining of lymphocytes and macrophages with CD150 in the lymph node, (IF) Bar: 50 µm. (**Hc**) Nectin-4-negative reaction in the lymph node, (IF) Bar: 50 µm. (**Hd**) Merged image of CDV, CD150, and Nectin-4 in the lymph node, resulting in CDV- and CD150-positive lymphocytes and macrophages (IF). Bar: 50 µm. (**He**) H&E-stained image of the same section subjected to IF in the lymph node, (H&E) Bar: 50 µm. (**Hf**) CDV-positive lymphocytes and macrophages in the section stained with H&E in the lymph node, (H&E + IF) Bar: 50 µm.

**Figure 8:**
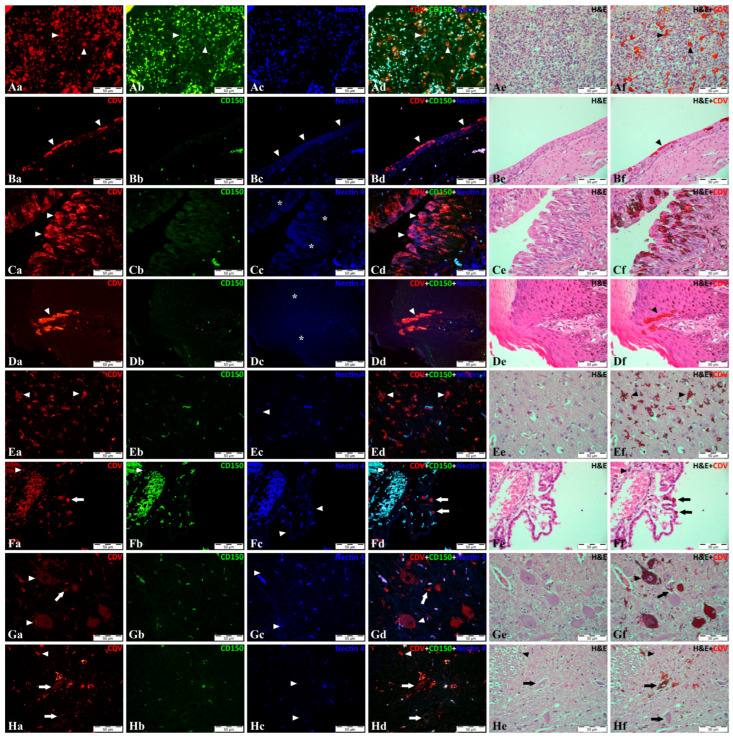
Triple IF images for CDV, CD150, and Nectin-4 in organs and tissues of the dogs within the scope of the study. (**A**) Triple IF staining images for CDV, CD150, and Nectin-4 in the spleen. (**Aa**) CDV-positive reaction in lymphocytes and macrophages in the white pulp (arrowheads), (IF) Bar: 50 µm. (**Ab**) CD150-positive lymphocytes and macrophages in the white pulp (arrowheads), (IF) Bar: 50 µm. (**Ac**) Nectin-4-negative reaction in the white pulp, (IF) Bar: 50 µm. (**Ad**) Merged image of CDV, CD150, and Nectin-4 in the spleen, resulting in CD150- and CDV-positive lymphocytes and macrophages (arrowheads), (IF) Bar: 50 µm. (**Ae**) H&E-stained image of the same section subjected to IF in the spleen, (H&E) Bar: 50 µm. (**Af**) CDV-positive lymphocytes and macrophages in the section stained with H&E (arrowheads), (H&E + IF) Bar: 50 µm. (**B**) Triple IF staining images for CDV, CD150, and Nectin-4 in the renal pelvis in the kidney. (**Ba**) CDV-positive reaction in renal pelvis epithelia (arrowheads), (IF) Bar: 50 µm. (**Bb**) CD150-negative reaction in the renal pelvis, (IF) Bar: 50 µm. (**Bc**) Nectin-4 positive reaction in renal pelvis epithelia (arrowheads), (IF) Bar: 50 µm. (**Bd**) Merged image of CDV, CD150, and Nectin-4 in the renal pelvis; Nectin-4 and CDV-positive reaction in renal pelvis epithelia (arrowheads), (IF) Bar: 50 µm. (**Be**) H&E-stained image of the same section subjected to IF in the renal pelvis, (H&E) Bar: 50 µm. (**Bf**) CDV-positive reaction in renal pelvis epithelia in the section stained with H&E (arrowhead), (H&E + IF) Bar: 50 µm. (**C**) Triple IF staining images for CDV, CD150, and Nectin-4 in the urinary bladder. (**Ca**) CDV-positive reaction in urinary bladder mucosal epithelial cells (arrowheads), (IF) Bar: 50 µm. (**Cb**) CD150-negative staining in mucosal epithelia, (IF) Bar: 50 µm. (**Cc**) Nectin-4 positive reaction in urinary bladder mucosal epithelia (stars), (IF) Bar: 50 µm. (**Cd**) Merged image of CDV, CD150, and Nectin-4, resulting in CDV and Nectin-4 positive mucosal epithelia (arrowheads), (IF) Bar: 50 µm. (**Ce**) H&E-stained image of the same section subjected to IF in the urinary bladder, (H&E) Bar: 50 µm. (**Cf**) CDV-positive mucosal epithelia in the section stained with H&E (stars), (H&E + IF) Bar: 50 µm. (**D**) Triple IF staining images for CDV, CD150, and Nectin-4 in the planum nasale. (**Da**) CDV-positive reaction in epidermal keratinocytes (arrowhead), (IF) Bar: 50 µm. (**Db**) CD150-negative staining in the epidermis in the planum nasale, (IF) Bar: 50 µm. (**Dc**) Nectin-4 positive keratinocytes in the epidermal layer (stars), (IF) Bar: 50 µm. (**Dd**) Merged image of CDV, CD150, and Nectin-4, resulting in Nectin-4 and CDV-positive epidermal keratinocytes (arrowhead), (IF) Bar: 50 µm. (**De**) H&E-stained image of the same section subjected to IF, (H&E) Bar: 50 µm. (**Df**) CDV-positive reaction in the Malpighian layer in the section stained with H&E (arrowhead), (H&E + IF) Bar: 50 µm. (**E**) Triple IF staining images for CDV, CD150, and Nectin-4 in the brain. (**Ea**) CDV-positive astrocytes in the brain (arrowheads), (IF) Bar: 50 µm. (**Eb**) CD150-negative reaction in the brain, (IF) Bar: 50 µm. (**Ec**) Nectin-4 positive neuron (arrowhead), (IF) Bar: 50 µm. (**Ed**) Merged image of CDV, CD150, and Nectin-4, resulting in CDV-positive but Nectin-4 and CD150-negative astrocytes (arrowheads), (IF) Bar: 50 µm. (**Ee**) H&E-stained image of the same section subjected to IF, (H&E) Bar: 50 µm. (**Ef**) CDV-positive astrocytes in the section stained with H&E (arrowheads), (H&E + IF) Bar: 50 µm. (**F**) Triple IF staining images for CDV, CD150, and Nectin-4 in the fourth ventricle choroid plexus. (**Fa**) CDV-positive areas: lymphocyte (arrowhead) and choroid plexus epithelia (arrow), (IF) Bar: 50 µm. (**Fb**) CD150-positive lymphocyte (arrowhead), (IF) Bar: 50 µm. (**Fc**) Nectin-4 positive choroid plexus epithelia (arrowheads), (IF) Bar: 50 µm. (**Fd**) Merged image of CDV, CD150, and Nectin-4; CD150- and CDV-positive lymphocyte (arrowhead), and Nectin-4 and CDV-positive choroid plexus epithelial cells (arrow), (IF) Bar: 50 µm. (**Fe**) H&E-stained image of the same section subjected to IF, (H&E) Bar: 50 µm. (**Ff**) CDV-positive lymphocyte (arrowhead) and choroid plexus epithelia (arrow) in the section stained with H&E, (H&E + IF) Bar: 50 µm. (**G**) Triple IF staining images for CDV, CD150, and Nectin-4 in the brainstem. (**Ga**) CDV-positive motor neurons (arrowheads) and astrocytes (arrow), (IF) Bar: 50 µm. (**Gb**) CD150-negative reaction, (IF) Bar: 50 µm. (**Gc**) Nectin-4 positive motor neurons (arrowheads), (IF) Bar: 50 µm. (**Gd**) Merged image of CDV, CD150, and Nectin-4, resulting in Nectin-4 and CDV-positive motor neurons (arrowhead), and CDV-positive but Nectin-4- and CD150-negative astrocytes (arrow), (IF) Bar: 50 µm. (**Ge**) H&E-stained image of the same section subjected to IF in the brainstem, (H&E) Bar: 50 µm. (**Gf**) CDV-positive motor neurons (arrowhead) and astrocytes (arrow) in the section stained with H&E in the brainstem, (H&E + IF) Bar: 50 µm. (**H**) Triple IF staining images for CDV, CD150, and Nectin-4 in the cervical spinal cord. (**Ha**) CDV-positive motor neuron (arrow) and astrocytes (arrowhead), (IF) Bar: 50 µm. (**Hb**) CD150-negative staining, (IF) Bar: 50 µm. (**Hc**) Nectin-4 positive motor neuron (arrowhead), (IF) Bar: 50 µm. (**Hd**) Merged image of IF staining for CDV, CD150, and Nectin-4, resulting in Nectin-4 and CDV-positive motor neuron (arrow), and CDV-positive but CD150- and Nectin-4-negative astrocyte (arrowhead), (IF) Bar: 50 µm. (**He**) Intracytoplasmic inclusion bodies in the motor neuron (arrow) and intranuclear inclusion bodies in the astrocyte (arrowhead) in the H&E-stained image of the same section subjected to IF in the cervical spinal cord, (H&E) Bar: 50 µm. (**Hf**) CDV-positive motor neuron (arrow) and astrocyte (arrowhead) in the section stained with H&E in the cervical spinal cord, (H&E + IF) Bar: 50 µm.

**Figure 9:**
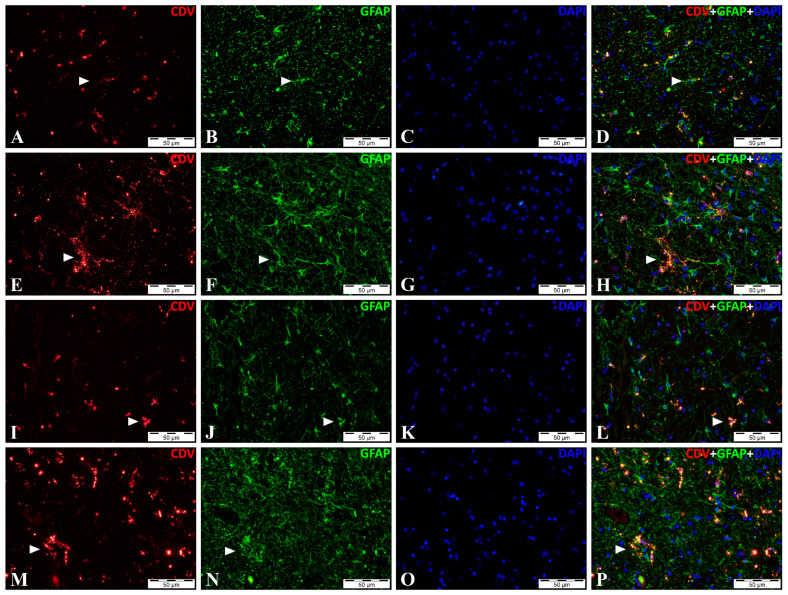
Double IF images for CDV and GFAP in the central nervous system. (**A**) CDV-positive areas in the brain parenchyma (arrowhead), (IF) Bar: 50 µm. (**B**) GFAP-positive reaction in astrocytes in the brain (arrowhead), (IF) Bar: 50 µm. (**C**) Nuclear DAPI staining in the brain, (IF) Bar: 50 µm. (**D**) Merged image of CDV, GFAP, and DAPI in the brain, resulting in CDV- and GFAP-positive astrocytes (arrowhead), (IF) Bar: 50 µm. (**E**) CDV-positive areas in the cerebellum (arrowhead), (IF) Bar: 50 µm. (**F**) GFAP-positive reaction in astrocytes in the cerebellum (arrowhead), (IF) Bar: 50 µm. (**G**) Nuclear DAPI staining in the cerebellum, (IF) Bar: 50 µm. (**H**) Merged image of CDV, GFAP, and DAPI in the cerebellum, resulting in CDV- and GFAP-positive astrocytes (arrowhead), (IF) Bar: 50 µm. (**I**) CDV-positive areas in the brainstem tissue (arrowhead), (IF) Bar: 50 µm. (**J**) GFAP-positive areas in astrocytes in the brainstem tissue (arrowhead), (IF) Bar: 50 µm. (**K**) Nuclear DAPI staining in the brainstem tissue, (IF) Bar: 50 µm. (**L**) Merged image of CDV, GFAP, and DAPI in the brainstem tissue, resulting in CDV- and GFAP-positive astrocytes (arrowhead), (IF) Bar: 50 µm. (**M**) CDV-positive reaction in the spinal cord (arrowhead), (IF) Bar: 50 µm. (**N**) GFAP-positive areas in the spinal cord (arrowhead), (IF) Bar: 50 µm. (**O**) Nuclear DAPI staining in the spinal cord, (IF) Bar: 50 µm. (**P**) Merged image of CDV, GFAP, and DAPI in the spinal cord, resulting in CDV- and GFAP-positive astrocytes (arrowhead), (IF) Bar: 50 µm.

**Table 1:** Primary and Secondary Antibodies.

Primary and Secondary Antibodies			
Antibodies	Antibodies Code	Incubation	Dilutions
Monoclonal Mouse anti CDV	MA1-82327	6 °C Overnight	1/1500
Human Nectin 4 Polyclonal Antibody	AF2659	6 °C Overnight	1/20
Rabbit anti-SLAM/CD150 Polyclonal antibody	10837-R008	6 °C Overnight	1/500
Rabbit Anti-Glial Fibrillary Acidic Protein (GFAP) Polyclonal Antibody	AB5804	6 °C Overnight	1/2000
Donkey anti-Goat IgG (H+L) Secondary Antibody, Alexa Fluor 350	A 21081	1 h. Room temperature	1/500
Goat anti-Rabbit IgG (H+L) Secondary Antibody, Alexa Fluor 488	A-11008	1 h. Room temperature	1/500
Goat anti-Mouse IgG (H+L) Secondary Antibody, Alexa Fluor 555	A-21422	1 h. Room temperature	1/500
Biotinylated Rabbit anti-Goat IgG (H+L) Secondary Antibody	31732	1 h. Room temperature	1/500
Normal Rabbit Serum		30 min. Room temperature	1/100
Normal Donkey Serum		30 min. Room temperature	1/100
Normal Goat Serum		30 min. Room temperature	1/100

**Table 2:** Case Information, Clinical Findings, IC Results, and RT-PCR Results.

Case No	Breed	Age	Sex	Neurological Symptoms	Respiratory Problems	Nasal and Ocular Discharge	IC Results	RT-PCR Results
1	Mongrel	>1 years old	Female	+	+	+	-	-
2	Mongrel	6–12 Month	Female	+	+	+	+	+
3	Mongrel *	1 Month	Male	-	+	+	+	+
4	Mongrel	4–5 Month	Female	+	+	+	-	+
5	Mongrel	4–5 Month	Male	+	+	-	+	+
6	Mongrel	3 Month	Female	+	+	+	+	+
7	Mongrel *	1.5 Month	Female	-	+	+	+	+
8	Mongrel *	2.5 Month	Male	-	+	+	+	+
9	Mongrel *	1.5–2 Month	Female	-	+	+	+	+
10	Mongrel *	1.5–2 Month	Female	-	+	+	+	+
11	Mongrel *	1.5–2 Month	Female	-	+	+	+	+
12	Mongrel *	3 Month	Male	-	+	+	+	+
13	Mongrel *	3 Month	Female	-	+	+	+	+
14	Mongrel *	3 Month	Male	-	+	+	+	+
15	Mongrel *	2.5–3 Month	Female	-	+	+	+	+
16	Mongrel *	3 Month	Male	-	+	+	+	+
17	Mongrel *	3–3.5 Month	Male	-	+	+	+	+
18	Mongrel *	3.5 Month	Male	-	+	+	+	+
19	Mongrel *	2.5 Month	Female	-	+	+	+	+
20	Mongrel	3 Month	Female	-	+	+	+	+
21	Mongrel	3 Month	Female	+	+	+	+	+
22	Mongrel	9 Month	Female	+	+	+	+	+
23	Mongrel	+1 Age	Female	+	+	+	-	-
24	Mongrel	2 Month	Female	+	+	+	ND	+
25	Mongrel	2 Month	?	+	?	+	ND	+
26	Mongrel	3 Month	?	+	+	+	ND	+
27	?	?	?	+	?	?	ND	+
28	Mongrel	>1 years old	Female	+	-	+	ND	+
29	Mongrel	2–3 Month	Female	+	?	+	ND	+
30	Mongrel	4 Month	?	+	?	+	ND	+
31	Mongrel	8 Month	Female	+	?	?	ND	+
32	Mongrel	4–5 Month	Male	+	?	+	ND	+
33	?	?	?	+	?	?	ND	+
34	Mongrel	2.5 Month	Male	+	?	?	ND	+

-: Negative; +: Positive; ?: Unknown due to the lack of official medical records for stray dogs; ND: Not Done (Archived Material); *: Orphaned or early-separated puppies reared by surrogate nursing, with suspected inadequate passive antibody transfer.

**Table 3:** CDV Immunohistochemical Staining Results.

Case No	Organs and Tissues																				
	Lungs	Heart	Mediastinal and Tracheobronchial Lymph Nodes	Thymus	Liver	Stomach	Pancreas	Duodenum	Jejunum	Ileum	Mesenteric Lymph Nodes	Spleen	Kidney	Bladder	Brain	Cerebellum	Brainstem	Spinal Cord	Footpad	Planum Nasale	Blood
1	-	-	-	-	-	-	-	-	-	-	-	-	-	-	-	-	-	-	0	0	-
2	++	-	+	0	+	0	+	+	+	++	+++	+	-	-	+	+	++	++	0	+	-
3	+++	-	+	++	+	+	+	+	+	++	++	++	+	+++	+	-	+	+	+	+++	-
4	-	-	+	-	-	+	+	-	-	-	-	+	-	-	+	+	+	+	0	-	-
5	++	-	+	++	+	+	+	-	-	+	++	++	+	+	+	++	+	+	+	+	-
6	++	-	++	++	-	+	+	-	-	-	-	-	+	+	++	++	++	++	+++	+	-
7	+	-	+	+++	-	+	-	+	+	++	+	++	-	-	+	-	-	-	-	-	-
8	+++	+	+++	+++	++	+	+	+	++	++	+++	+++	+	+++	+	+	+	+	++	++	-
9	+++	-	+++	+++	+	+	+	+	+	++	+++	+++	++	++	+	+	+	+	-	-	-
10	++	-	+++	+++	+	+	+	+	+	++	+++	++	+	+	+	-	-	+	-	+	-
11	++	-	++	+++	+++	+++	+	+	+	++	+++	+++	+	+	++	+	+	++	-	+	-
12	++	-	+	+	+++	+	+	+	+	++	++	+	+	+	-	+	-	-	+	+	+
13	++	-	+	0	+	++	-	0	-	+	+	+	+	+	+	+	+	+	+	+	-
14	-	-	+	+	+	-	-	-	+	+	+	+	-	-	-	+	+	+	-	-	-
15	+	-	++	+	+	+	+	+	+	++	0	++	+	+	-	+	+	+	-	+	-
16	+	-	+	+	+	+	-	+	+	++	+	+	+	+	-	+	+	+	+	+	-
17	+	-	++	++	+	+	+	+	+	++	+++	++	+	+	+	-	-	+	-	-	-
18	-	-	++	+	-	+	+	+	+	+++	+	+	-	-	-	+	-	-	-	-	-
19	+	-	++	++	-	+	+	+	0	0	0	+	++	+	-	-	-	-	0	-	-
20	++	-	+	+	+	+	+	+	+	+	+	+	+	+++	+	+++	+++	+	+	+	-
21	++	-	+	0	+	+	+	+	+	+	+	+	+	+	+	+	+	+	+	+	-
22	+	-	-	-	-	-	-	-	-	-	-	-	+	-	+	+	+	+	+	-	-
23	-	-	-	-	-	-	-	-	-	-	-	-	-	-	-	-	-	-	-	-	-
24	+++	-	0	0	+++	0	+	+	+	0	0	0	+	0	+	+	+	0	0	0	+
25	++	-	0	0	+++	0	+	+	+	+	0	0	+	0	0	+	+	0	0	0	+
26	++	0	0	0	-	+	-	-	-	-	0	0	+	0	+	+	+	0	0	0	-
27	++	-	0	0	-	0	-	-	+	+	0	-	+	0	+	+	+	0	+	0	-
28	-	-	0	0	-	+	0	0	-	-	0	-	-	0	+	+	++	0	0	0	-
29	-	-	0	0	-	-	-	-	-	-	0	0	-	0	+	+	-	0	-	-	-
30	+	-	0	0	-	+	-	-	-	-	0	-	+	+	+	+	+	0	+	+	-
31	+	-	0	0	-	+	+	+	+	++	0	+	+	+	+	+	+	0	+	+	-
32	+	-	0	0	0	0	0	-	-	-	0	-	-	-	+	+	+	0	+	0	-
33	+++	-	0	0	0	0	++	-	-	+	0	-	+	+	+	+	+	0	0	0	-
34	+	0	0	0	+	+	-	-	-	+	0	+	+	+	+	+	+	0	+	-	-

-: Negative; +: Mild Positive; ++: Moderate Positive; +++: Severe Positive; 0: Could not be evaluated.

**Table 4:** CD150 Immunohistochemical Staining Results.

Organ	Immunopositive Reaction
**Lungs**	Alveolar macrophages
**Heart**	Negative
**Thymus**	Lymphocytes
**Spleen**	Lymphocytes and macrophages
**Lymph Nodes**	Lymphocytes and sinusoidal macrophages
**Stomach**	Infiltrative lymphocytes and macrophages
**Duodenum**	Infiltrative lymphocytes and macrophages
**Jejunum**	Infiltrative lymphocytes and macrophages
**Ileum**	Infiltrative lymphocytes and macrophages with Peyer’s patches (lymphocytes)
**Liver**	Kupffer cells
**Pancreas**	Negative
**Renal pelvis**	Infiltrative lymphocytes and macrophages
**Bladder**	Few lymphocytes and macrophages in the lamina propria
**Footpad and Planum Nasale**	Infiltrative lymphocytes and macrophages
**Brain**	Microglial cells and few lymphocytes
**Cerebellum**	Microglial cells and few lymphocytes
**Brainstem**	Microglial cells and few lymphocytes
**Spinal Cord**	Microglial cells and few lymphocytes

**Table 5:** Nectin-4 Immunohistochemical Staining Results.

Organ	Immunopositive Reaction
**Lungs**	Bronchial and bronchiolar epithelia
**Heart**	Negative
**Thymus**	Negative
**Liver**	Bile duct epithelia
**Spleen**	Negative
**Lymph Nodes**	Negative
**Stomach**	Mucosal surface epithelia and parietal cells
**Duodenum**	Villus and crypt epithelia
**Jejunum**	Villus and crypt epithelia
**Ileum**	Villus and crypt epithelia
**Pancreas**	Non-specific staining
**Kidney**	Renal pelvis and some tubular epithelia
**Bladder**	Mucosal epithelium
**Footpad and Planum Nasale**	Epidermal layer cells and sweat gland epithelia in the dermis
**Brain**	Ependymal cells, choroid plexus epithelia, and some neurons.
**Cerebellum**	Ependymal cells, choroid plexus epithelia, and some neurons.
**Brainstem**	Motor neurons
**Spinal Cord**	Motor neurons and central canal ependymal cells

## Data Availability

The original contributions presented in this study are included in the article. Further inquiries can be directed to the corresponding author.

## Abbreviations

The following abbreviations are used in this manuscript:

µL	Microliter
µm	Micrometer
+	Positive
-	Negative
?	Unknown
0	Not evaluated
CD	Canine Distemper
CDV	Canine Distemper Virus
CSF	Cerebrospinal Fluid
CNS	Central Nervous System
DNA	Deoxyribonucleic Acid
GFAP	Glial Fibrillary Acidic Protein
H&E	Hematoxylin-Eosin
IC	Immunochromatography
IHC	Immunohistochemistry
IF	Immunofluorescence
*L. epithelialis*	*Lamina epithelialis*
*L. propria*	*Lamina propria*
MV	Measles Virus
N4	Nectin-4
ND	Not Done (Archived Material)
nt	Nucleotide
PBS	Phosphate Buffer Solution
pH	Power of Hydrogen
RT-PCR	Reverse-Transcriptase Polymerase Chain Reaction
SLAM	Signaling Lymphocyte Activation Molecule
SLAMF1	Signaling Lymphocyte Activation Molecule Family 1
UV	Ultraviolet

## References

[1] Vandevelde M., Higgins R.J., Kristensen B., Kristensen F., Steck A.J., Kihm U. (1982). Demyelination in experimental canine distemper virus infection: Immunological, pathologic, and immunohistological studies. Acta Neuropathol..

[2] Barrett T. (1999). Morbillivirus infections, with special emphasis on morbilliviruses of carnivores. Vet. Microbiol..

[3] Koutinas A.F., Polizopoulou Z.S., Baumgaertner W., Lekkas S., Kontos V. (2002). Relation of clinical signs to pathological changes in 19 cases of canine distemper encephalomyelitis. J. Comp. Pathol..

[4] Esin E. (2013). Köpeklerde Canine Distemper Virus Enfeksiyonunun Araştırılması. Doctoral Dissertation.

[5] Lempp C., Spitzbarth I., Puff C., Cana A., Kegler K., Techangamsuwan S., Baumgärtner W., Seehusen F. (2014). New aspects of the pathogenesis of canine distemper leukoencephalitis. Viruses.

[6] Martinez-Gutierrez M., Ruiz-Saenz J. (2016). Diversity of susceptible hosts in canine distemper virus infection: A systematic review and data synthesis. BMC Vet. Res..

[7] Silva M.D.L., Silva G.E.B., Borin-Crivellenti S., Alvarenga A.W.O., Aldrovani M., do Nascimento Braz L.A., Aoki C., Santana A.E., Pennacchi C.S., Crivellenti L.Z. (2022). Renal Abnormalities Caused by Canine Distemper Virus Infection in Terminal Patients. Front. Vet. Sci..

[8] Tatsuo H., Ono N., Tanaka K., Yanagi Y. (2000). SLAM (CDw150) is a cellular receptor for measles virus. Nature.

[9] Noyce R.S., Bondre D.G., Ha M.N., Lin L.T., Sisson G., Tsao M.S., Richardson C.D. (2011). Tumor cell marker PVRL4 (Nectin 4) is an epithelial cell receptor for measles virus. PLoS Pathog..

[10] Zhao J., Ren Y. (2022). Multiple Receptors Involved in Invasion and Neuropathogenicity of Canine Distemper Virus: A Review. Viruses.

[11] Rendon-Marin S., da Fontoura Budaszewski R., Canal C.W., Ruiz-Saenz J. (2019). Tropism and molecular pathogenesis of canine distemper virus. Virol. J..

[12] Alves L., Khosravi M., Avila M., Ader-Ebert N., Bringolf F., Zurbriggen A., Vandevelde M., Plattet P. (2015). SLAM- and nectin-4-independent noncytolytic spread of canine distemper virus in astrocytes. J. Virol..

[13] Takeda M., Seki F., Yamamoto Y., Nao N., Tokiwa H. (2020). Animal morbilliviruses and their cross-species transmission potential. Curr. Opin. Virol..

[14] Sambrook J., Russell D.W. (2006). Purification of nucleic acids by extraction with phenol: Chloroform. Cold Spring Harb. Protoc..

[15] Frisk A.L., König M., Moritz A., Baumgärtner W. (1999). Detection of canine distemper virus nucleoprotein RNA by reverse transcription-PCR using serum, whole blood, and cerebrospinal fluid from dogs with distemper. J. Clin. Microbiol..

[16] Karakurt E., Beytut E., Dağ S., Nuhoğlu H., Yıldız A., Kurtbaş E. (2022). Assessment of MDA and 8-OHdG expressions in ovine pulmonary adenocarcinomas by immunohistochemical and immunofluorescence methods. Acta Vet. Brno.

[17] Tatsuo H., Ono N., Yanagi Y. (2001). Morbilliviruses use signaling lymphocyte activation molecules (CD150) as cellular receptors. J. Virol..

[18] Seki F., Ono N., Yamaguchi R., Yanagi Y. (2003). Efficient isolation of wild strains of canine distemper virus in Vero cells expressing canine SLAM (CD150) and their adaptability to marmoset B95a cells. J. Virol..

[19] Von Messling V., Oezguen N., Zheng Q., Vongpunsawad S., Braun W., Cattaneo R. (2005). Nearby clusters of hemagglutinin residues sustain SLAM-dependent canine distemper virus entry in peripheral blood mononuclear cells. J. Virol..

[20] Hashiguchi T., Ose T., Kubota M., Maita N., Kamishikiryo J., Maenaka K., Yanagi Y. (2011). Structure of the measles virus hemagglutinin bound to its cellular receptor SLAM. Nat. Struct. Mol. Biol..

[21] Romanets-Korbut O., Kovalevska L., Seya T., Sidorenko S.P., Horvat B. (2016). Measles virus hemagglutinin triggers intracellular signaling in CD150-expressing dendritic cells and inhibits immune response. Cell. Mol. Immunol..

[22] Dragovich M.A., Mor A. (2018). The SLAM family receptors: Potential therapeutic targets for inflammatory and autoimmune diseases. Autoimmun. Rev..

[23] Pratakpiriya W., Seki F., Otsuki N., Sakai K., Fukuhara H., Katamoto H., Hirai T., Maenaka K., Techangamsuwan S., Lan N.T. (2012). Nectin4 is an epithelial cell receptor for canine distemper virus and involved in neurovirulence. J. Virol..

[24] Delpeut S., Noyce R.S., Richardson C.D. (2014). The tumor-associated marker, PVRL4 (nectin-4): Is the epithelial receptor for morbilliviruses. Viruses.

[25] Zhao J., Shi N., Sun Y., Martella V., Nikolin V., Zhu C., Zhang H., Hu B., Bai X., Yan X. (2015). Pathogenesis of canine distemper virus in experimentally infected raccoon dogs, foxes, and minks. Antivir. Res..

[26] Sarchahi A.A., Arbabi M., Mohebalian H. (2022). Detection of canine distemper virus in cerebrospinal fluid, whole blood and mucosal specimens of dogs with distemper using RT-PCR and immunochromatographic assays. Vet. Med. Sci..

[27] Isvand M., Mokhtari A., Esmailnejad A. (2020). Genomic and Immunological Identification of Canine Distemper Virus (CDV) and Investigation of Coinfection with *Bordetella bronchiseptica* Among Dogs in Iran. Jentashapir J. Cell. Mol. Biol..

[28] Fischer C.D.B., Ikuta N., Canal C.W., Makiejczuk A., da Costa Allgayer M., Cardoso C.H., Lehmann F.K., Fonseca A.S.K., Lunge V.R. (2013). Detection and differentiation of field and vaccine strains of canine distemper virus using reverse transcription followed by nested real time PCR (RT-nqPCR) and RFLP analysis. J. Virol. Methods.

[29] Kim D., Jeoung S.Y., Ahn S.J., Lee J.H., Pak S.I., Kwon H.M. (2006). Comparison of tissue and fluid samples for the early detection of canine distemper virus in experimentally infected dogs. J. Vet. Med. Sci..

[30] Lan N.T., Yamaguchi R., Inomata A., Furuya Y., Uchida K., Sugano S., Tateyama S. (2006). Comparative analyses of canine distemper viral isolates from clinical cases of canine distemper in vaccinated dogs. Vet. Microbiol..

[31] Beineke A., Puff C., Seehusen F., Baumgärtner W. (2009). Pathogenesis and immunopathology of systemic and nervous canine distemper. Vet. Immunol. Immunopathol..

[32] Saunders W.B., Washabau R.J., Day M.J. (2013). Small Intestine. Canine and Feline Gastroenterology.

[33] da Fontoura Budaszewski R., Von Messling V. (2016). Morbillivirus experimental animal models: Measles virus pathogenesis insights from canine distemper virus. Viruses.

[34] Sheikh T.J., Ranjan R., Jha A.K., Kumar S. (2021). Canine distemper: A fatal disease seeking special intervention. J. Entomol. Zool. Stud..

[35] Higgins R.J., Krakowka S.G., Metzler A.E., Koestner A. (1982). Primary demyelination in experimental canine distemper virus induced encephalomyelitis in gnotobiotic dogs: Sequential immunologic and morphologic findings. Acta Neuropathol..

[36] Vandevelde M., Zurbriggen A., Higgins R.J., Palmer D. (1985). Spread and distribution of viral antigen in nervous canine distemper. Acta Neuropathol..

[37] Carvalho O.V., Botelho C.V., Ferreira C.G.T., Scherer P.O., Soares-Martins J.A.P., Almeida M.R., Silva Júnior A. (2012). Immunopathogenic and neurological mechanisms of canine distemper virus. Adv. Virol..

[38] Okita M., Yanai T., Ochikubo F., Gemma T., Mori T., Maseki T., Yamanouchi K., Mikami T., Kai C. (1997). Histopathological features of canine distemper recently observed in Japan. J. Comp. Pathol..

[39] Pardo I.D., Johnson G.C., Kleiboeker S.B. (2005). Phylogenetic characterization of canine distemper viruses detected in naturally infected dogs in North America. J. Clin. Microbiol..

[40] Headley S.A., Sukura A. (2009). Naturally occurring systemic canine distemper virus infection in a pup. Braz. J. Vet. Pathol..

[41] MacLachlan N.J., Dubovi E.J. (2011). Fenner’s Veterinary Virology.

[42] Batista Linhares M., Whiteley H.E., Samuelson J.P., Hsiao S.H., Stern A.W., Sprandel I.T., Roady P.J., Coleman D.A., Rizzo R., Froderman S.F. (2021). Sylvatic Canine Morbillivirus in Captive Panthera Highlights Viral Promiscuity and the Need for Better Prevention Strategies. Pathogens.

[43] Kaiser F.K., van Dyck L., Jo W.K., Schreiner T., Pfankuche V.M., Wohlsein P., Baumann I., Peters M., Baumgärtner W., Osterhaus A.D.M.E. (2021). Detection of Systemic Canine Kobuvirus Infection in Peripheral Tissues and the Central Nervous System of a Fox Infected with Canine Distemper Virus. Microorganisms.

[44] Headley S.A., Oliveira T.E., Pereira A.H., Moreira J.R., Michelazzo M.M., Pires B.G., Marutani V.H.B., Xavier A.A.C., Di Santis G.W., Garcia J.L. (2018). Canine morbillivirus (canine distemper virus) with concomitant canine adenovirus, canine parvovirus-2, and *Neospora caninum* in puppies: A retrospective immunohistochemical study. Sci. Rep..

[45] Machida N., Kiryu K., Oh-Ishi K., Kanda E., Izumisawa N., Nakamura T. (1993). Pathology and epidemiology of canine distemper in raccoon dogs (*Nyctereutes procyonoides*). J. Comp. Pathol..

[46] Chinnadurai S.K., Kinsel M.J., Adkesson M.J., Terio K. (2017). Canine distemper in a vaccinated snow leopard (*Panthera uncia*). J. Zoo Wildl. Med..

[47] Lan N.T., Yamaguchi R., Kien T.T., Hirai T., Hidaka Y., Nam N.H. (2009). First isolation and characterization of canine distemper virus in Vietnam with the immunohistochemical examination of the dog. J. Vet. Med. Sci..

[48] Krakowka S., Koestner A. (1976). Age-related susceptibility to infection with canine distemper virus in gnotobiotic dogs. J. Infect. Dis..

[49] Kadam R.G., Karikalan M., Siddappa C.M., Mahendran K., Srivastava G., Rajak K.K., Bhardwaj Y., Varshney R., War Z.A., Ghosh M. (2022). Molecular and pathological screening of canine distemper virus in Asiatic lions, tigers, leopards, snow leopards, clouded leopards, leopard cats, jungle cats, civet cats, fishing cat, and jaguar of different states, India. Infect. Genet. Evol..

[50] Alves C.M., Vasconcelos A.C., Martins A.S., Del Puerto H.L., Santos F.G.A., Nunes J.E.S., Campos P.P., Moro L. (2006). Morphometric analysis of the thymus of puppies infected with the Snyder Hill Strain of canine distemper virus. Arq. Bras. Med. Vet. Zootec..

[51] Iwatsuki K., Okita M., Ochikubo F., Gemma T., Shin Y.S., Miyashita N., Mikami T., Kai C. (1995). Immunohistochemical analysis of the lymphoid organs of dogs naturally infected with canine distemper virus. J. Comp. Pathol..

[52] Pope J.P., Miller D.L., Riley M.C., Anis E., Wilkes R.P. (2016). Characterization of a novel canine distemper virus causing disease in wildlife. J. Vet. Diagn. Investig..

[53] Liang C.T., Chueh L.L., Pang V.F., Zhuo Y.X., Liang S.C., Yu C.K., Chiang H., Liu C.H. (2007). A non-biotin polymerized horseradish-peroxidase method for the immunohistochemical diagnosis of canine distemper. J. Comp. Pathol..

[54] Takayama I., Kubo M., Takenaka A., Fujita K., Sugiyama T., Arai T., Yoneda M., Sato H., Yanai T., Kai C. (2009). Pathological and phylogenetic features of prevalent canine distemper viruses in wild masked palm civets in Japan. Comp. Immunol. Microbiol. Infect. Dis..

[55] Viana N.E., de Mello Zanim Michelazzo M., Oliveira T.E.S., Cubas Z.S., de Moraes W., Headley S.A. (2020). Immunohistochemical identification of antigens of canine distemper virus in neotropical felids from Southern Brazil. Transbound. Emerg. Dis..

[56] Megid J., Teixeira C.R., Amorin R.L., Cortez A., Heinemann M.B., de Paula Antunes J.M.A., da Costa L.F., Fornazari F., Cipriano J.R.B., Cremasco A. (2010). First identification of canine distemper virus in hoary fox (*Lycalopex vetulus*): Pathologic aspects and virus phylogeny. J. Wildl. Dis..

[57] Maeda H., Ozaki K., Takagi Y., Sawashima K., Narama I. (1994). Distemper skin lesions in a dog. J. Vet. Med. Ser. A.

[58] Areco W.V., Aguiar A., Barraza V., Fighera R.A., Kommers G., Flores M.M., Flores E.F. (2022). Macroscopic Distribution, Histopathology and Viral Antigen Expression in Dogs with Canine Distemper Virus-induced Hyperkeratosis in Nasodigital and Other Regions. J. Comp. Pathol..

[59] Koutinas A.F., Baumgärtner W., Tontis D., Polizopoulou Z., Saridomichelakis M.N., Lekkas S. (2004). Histopathology and immunohistochemistry of canine distemper virus-induced footpad hyperkeratosis (hard pad disease) in dogs with natural canine distemper. Vet. Pathol..

[60] Summers B.A., Greisen H.A., Appel M.J.G. (1984). Canine distemper encephalomyelitis: Variation with virus strain. J. Comp. Pathol..

[61] Krakowka S., Mador R.A., Koestner A. (1978). Canine distemper virus-associated encephalitis: Modification by passive antibody administration. Acta Neuropathol..

[62] Haines D.M., Martin K.M., Chelack B.J., Sargent R.A., Outerbridge C.A., Clark E.G. (1999). Immunohistochemical detection of canine distemper virus in haired skin, nasal mucosa, and footpad epithelium: A method for antemortem diagnosis of infection. J. Vet. Diagn. Investig..

[63] Damián M., Morales E., Salas G., Trigo F.J. (2005). Immunohistochemical detection of antigens of distemper, adenovirus and parainfluenza viruses in domestic dogs with pneumonia. J. Comp. Pathol..

[64] Michelazzo M.D.M.Z., de Oliveira T.E.S., Viana N.E., de Moraes W., Cubas Z.S., Headley S.A. (2020). Immunohistochemical evidence of canine morbillivirus (canine distemper) infection in coatis (*Nasua nasua*) from Southern Brazil. Transbound. Emerg. Dis..

[65] Oleaga Á., Vázquez C.B., Royo L.J., Barral T.D., Bonnaire D., Armenteros J.Á., Rabanal B., Gortázar C., Balseiro A. (2022). Canine distemper virus in wildlife in south-western Europe. Transbound. Emerg. Dis..

[66] Kabak Y.B., Sözmen M., Yarım M., Guvenç T., Karayiğit O.M., Gülbahar M.Y. (2015). Immunohistochemical detection of autophagy-related microtubule-associated protein 1 light chain 3 (LC3) in the cerebellums of dogs naturally infected with canine distemper virus. Biotech. Histochem..

[67] Palmer D.G., Huxtable C.R.R., Thomas J.B. (1990). Immunohistochemical demonstration of canine distemper virus antigen as an aid in the diagnosis of canine distemper encephalomyelitis. Res. Vet. Sci..

[68] Mutinelli F., Vandevelde M., Griot C., Richard A. (1989). Astrocytic infection in canine distemper virus-induced demyelination. Acta Neuropathol..

[69] Krakowka S. (1989). Canine distemper virus infectivity of various blood fractions for central nervous system vasculature. J. Neuroimmunol..

[70] Rudd P.A., Cattaneo R., Von Messling V. (2006). Canine distemper virus uses both the anterograde and the hematogenous pathway for neuroinvasion. J. Virol..

[71] Zachary J.F. (2017). Pathologic Basis of Veterinary Disease.

[72] Poelaert K.C., Williams R.M., Matullo C.M., Rall G.F. (2021). Noncanonical transmission of a measles virus vaccine strain from neurons to astrocytes. Mbio.

[73] Sato Y., Watanabe S., Fukuda Y., Hashiguchi T., Yanagi Y., Ohno S. (2018). Cell-to-cell measles virus spread between human neurons is dependent on hemagglutinin and hyperfusogenic fusion protein. J. Virol..

[74] Pratakpiriya W., Ping Teh A.P., Radtanakatikanon A., Pirarat N., Thi Lan N., Takeda M., Techangamsuwan S., Yamaguchi R. (2017). Expression of canine distemper virus receptor Nectin-4 in the central nervous system of dogs. Sci. Rep..

[75] Bleharski J.R., Niazi K.R., Sieling P.A., Cheng G., Modlin R.L. (2001). Signaling lymphocytic activation molecule is expressed on CD40 ligand-activated dendritic cells and directly augments production of inflammatory cytokines. J. Immunol..

[76] Wenzlow N., Plattet P., Wittek R., Zurbriggen A., Grone A. (2007). Immunohistochemical demonstration of the putative canine distemper virus receptor CD150 in dogs with and without distemper. Vet. Pathol..

[77] Castro A.G., Hauser T.M., Cocks B.G., Abrams J., Zurawski S., Churakova T., Zonin F., Robinson D., Tangye S.G., Aversa G. (1999). Molecular and functional characterization of mouse signaling lymphocytic activation molecule (SLAM): Differential expression and responsiveness in Th1 and Th2 cells. J. Immunol..

[78] Garcia P.A.T., Cartoceti A., Affolter V.K., Jackson K., Keel M.K., Agnew D., Cooley T., Melotti J., Fitzgerald D.T., Pesavento P.A. (2022). Distribution of canine distemper virus and nectin-4 in raccoon (*Procyon lotor*) skin. Vet. Pathol..

[79] Francesca V., Sandra S., Alessandra S., Gualtiero G., Luciana G., Laura C. (2006). A molecular study of hippocampus in dogs with convulsion during canine distemper virus encephalitis. Brain Res..

